# Geminin deficiency enhances survival in a murine medulloblastoma model by inducing apoptosis of preneoplastic granule neuron precursors

**DOI:** 10.18632/genesandcancer.157

**Published:** 2017-09

**Authors:** Savita Sankar, Ethan Patterson, Emily M. Lewis, Laura E. Waller, Caili Tong, Joshua Dearborn, David Wozniak, Joshua B. Rubin, Kristen L. Kroll

**Affiliations:** ^1^ Department of Developmental Biology, Washington University School of Medicine, Saint Louis, MO, USA; ^2^ Department of Psychiatry, Washington University School of Medicine, Saint Louis, MO, USA; ^3^ Department of Pediatrics, Washington University School of Medicine, Saint Louis, MO, USA

**Keywords:** neural, medulloblastoma, cerebellum, DNA replication, apoptosis

## Abstract

Medulloblastoma is the most common malignant brain cancer of childhood. Further understanding of tumorigenic mechanisms may define new therapeutic targets. Geminin maintains genome fidelity by controlling re-initiation of DNA replication within a cell cycle. In some contexts, Geminin inhibition induces cancer-selective cell cycle arrest and apoptosis and/or sensitizes cancer cells to Topoisomerase IIα inhibitors such as etoposide, which is used in combination chemotherapies for medulloblastoma. However, Geminin's potential role in medulloblastoma tumorigenesis remained undefined. Here, we found that Geminin is highly expressed in human and mouse medulloblastomas and in murine granule neuron precursor (GNP) cells during cerebellar development. Conditional Geminin loss significantly enhanced survival in the SmoA1 mouse medulloblastoma model. Geminin loss in this model also reduced numbers of preneoplastic GNPs persisting at one postnatal month, while at two postnatal weeks these cells exhibited an elevated DNA damage response and apoptosis. Geminin knockdown likewise impaired human medulloblastoma cell growth, activating G2 checkpoint and DNA damage response pathways, triggering spontaneous apoptosis, and enhancing G2 accumulation of cells in response to etoposide treatment. Together, these data suggest preneoplastic and cancer cell-selective roles for Geminin in medulloblastoma, and suggest that targeting Geminin may impair tumor growth and enhance responsiveness to Topoisomerase IIα-directed chemotherapies.

## INTRODUCTION

Initiation of DNA replication is a tightly controlled process, involving formation of pre-replication complexes (pre-RCs) on sites in the genome [1-3]. After initiation of DNA replication, several convergent pathways restrict additional pre-RC formation, limiting genome duplication to once per cell division [4-9]. The primary target of this regulation in most metazoan cells is Cdt1, which is inactivated by mechanisms involving either Cdt1 proteolysis or inhibitory interaction with the nucleoprotein Geminin (Gmnn) [1, 2, 4, 7, 9]. Gmnn-mediated inhibition of Cdt1 becomes active during S/G2 to prevent reinitiation of DNA replication within a cell cycle, while Gmnn proteolysis during mitosis enables initiation of a new round of replication in the subsequent cell cycle [9]. Cells bypassing these controls accumulate DNA damage, including >4N DNA content and stalled replication forks. This can trigger G2 checkpoint arrest, which is frequently followed by apoptosis.

Prior work comparing Gmnn requirements in multiple cancer- and non-cancer-derived cell lines suggested that some cancer cell lines are particularly dependent upon levels of Gmnn activity and selectively sensitive to Gmnn inhibition, exhibiting re-replication, G2 checkpoint activation, and apoptosis under conditions where Gmnn-deficient non-cancer cell lines are unaffected [10]. This cancer cell sensitivity to Gmnn loss may involve greater relative ability of non-cancer cell lines to use alternate molecular mechanisms to control Cdt1 activity by proteolysis, and/or relative over-expression of Cdt1 and pre-RC proteins in many cancer cell contexts [10, 11]. Based upon these results, it has been proposed that small molecule inhibitors directed against Gmnn may have utility as selective anticancer agents [10, 12].

*Gmnn* is over-expressed in many tumor types, with high expression frequently serving as a diagnostic criterion for aggressiveness and poor prognosis [13-21]. In addition to a role in maintaining genome fidelity, Gmnn is required for several aspects of embryonic development, and can control embryonic gene expression through interactions with chromatin regulatory complexes [22-35]. For example, Gmnn promotes neural fate acquisition of embryonic stem cells [30, 36], while loss of Gmnn function in the forming central nervous system from embryonic day 8.0 (E8.0) in conditional mouse models results in neural tube defects, at least in part through failure to activate expression of genes that promote neural tube patterning and neuronal differentiation [29].

Given Gmnn's potential to selectively inhibit DNA replication in other types of cancer, we hypothesized that Gmnn could potentially modulate tumorigenesis in medulloblastoma, the most common malignant pediatric brain tumor and the leading cause of cancer-related death in children. Medulloblastoma accounts for ∼20% of all malignant brain cancers of childhood [37]. Multi-modal treatment including tumor resection, radiotherapy, and adjuvant chemotherapy have improved long term event-free survival for average risk patients, but outcomes are inferior in children of <3 years or in patients with tumor recurrence [37-39]. Further development of targeted treatments is likely to emerge from an improved understanding of the molecular mechanisms underlying this disease. Therefore, here we used both mouse animal and human cell models to study whether Gmnn could act as a modifier of medulloblastoma tumorigenesis and to begin to elucidate some of the underlying mechanisms.

## RESULTS

### *Geminin* is highly expressed in human and mouse medulloblastoma

**Figure 1:**
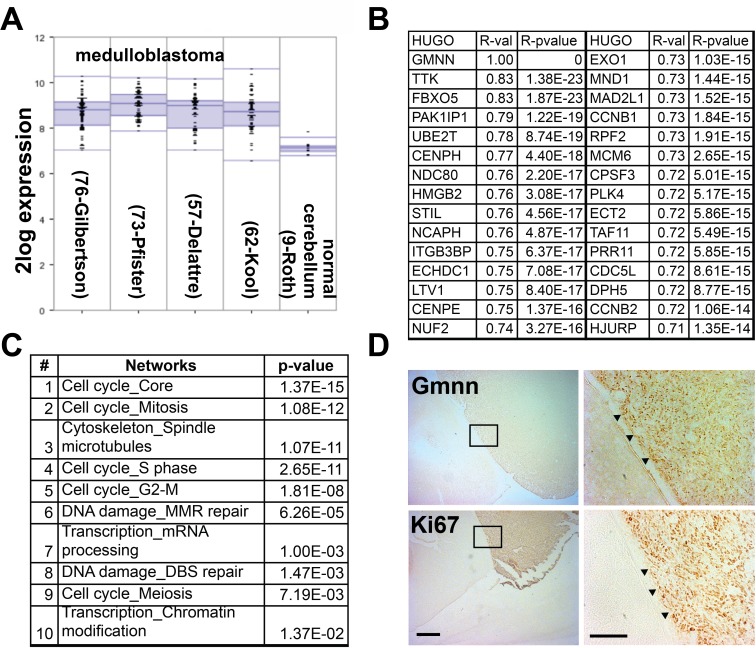
*Geminin* is highly expressed in human and mouse medulloblastoma (**A**) Elevated *Gmnn* expression was detected in four human medulloblastoma microarray datasets relative to normal cerebellum. (**B-C**) Top genes **(B)** and gene ontology (GO) terms (**C**) whose expression most strongly correlated with *Gmnn* expression were defined in 103 medulloblastoma samples (Northcott core transcript; GSE21140) using R2 (see Methods). (**D**) Gmnn and Ki67 immunostaining of cerebellar sections from an adult SmoA1 mouse with medulloblastoma. Boxed insets (right panels) at the tumor boundary (marked with arrowheads) show relative expression levels in normal cerebellum (left) versus tumor (right) for Gmnn and Ki67. Scale bars= 500μm (left) and 100μm (right).

As Gmnn inhibition selectively impaired the growth of several cancer cell lines under conditions where normal/non-cancer lines were not affected [10], we hypothesized that Gmnn inhibition might represent a therapeutic target inmedulloblastoma.Usingpubliclyavailabledata, we found that *Gmnn* expression is elevated in human medulloblastomas, relative to normal cerebellum (Figure 1A). Human tumors with high *Gmnn* expression levels also exhibit high levels of expression of genes associated with the cell cycle, DNA damage/repair, and components of the pre-replication complex (e.g. *Ccnb1/b2*, *Cenpe/h*, *Mcm6*, *Orc1/6*)(Figure 1B-1C; Supplementary Table 1). *Gmnn* expression was most strongly anti-correlated with terms associated with differentiated neural cells (transmission of nerve impulse, neuropeptide signaling, voltage-gated channel). All genes positively correlated with *Gmnn* in human medulloblastoma and correlated and anti-correlated GO terms are in Supplementary Tables 1-3. These data are consistent with *Gmnn* being most highly expressed in rapidly proliferating cells of the tumor and anti-correlated with differentiated cells or brain regions. Likewise, in a murine medulloblastoma model (SmoA1), Gmnn and the proliferative cell marker Ki-67 were both strongly expressed in tumor tissue, while neither marker was expressed in adjacent normal brain tissue (Figure 1D).

Based upon genomic sequencing and expression analysis, medulloblastomas have been divided into four major molecular subgroups: Wnt, Sonic Hedgehog (Shh), Group 3, and Group 4 [40-43]. We therefore examined *Gmnn* expression levels in human medulloblastomas based on molecular and histological subtypes, sex, and several other criteria (presence of a β-catenin mutation, staging, time of diagnosis), using two medulloblastoma expression datasets. No subtype or sex-dependent differences in *Gmnn* levels were seen, with the exception of lower *Gmnn* levels in Wnt subtype tumors with monosomy 6, consistent with the location of *Gmnn* on chromosome 6 (Supplementary Figure 1).

### *Geminin* deficiency enhances survival in the SmoA1 medulloblastoma model

About 30% of human medulloblastomas exhibit constitutive Shh pathway activation [44], resulting from either inactivating mutations in the negative regulators Patched1 (Ptch1) or Suppressor of Fused (Sufu), or from activating mutations in the signal transducing receptor Smoothened (Smo) or amplification of the Gli2 transcription factor [42, 45-47]. Formation of these tumors involves abnormal development of granule neuron precursors (GNPs) [48, 49]. Granule neurons are the most abundant cerebellar neurons and develop from committed GNPs, which migrate over the surface of the developing cerebellum to form the external granule layer (EGL) and undergo major expansion in the EGL during the first two postnatal weeks of development in the mouse [49, 50]. After this, they exit the cell cycle, differentiate, and migrate through the adjacent Purkinje cell layer to establish the internal granule layer (IGL) [48, 49].

**Figure 2:**
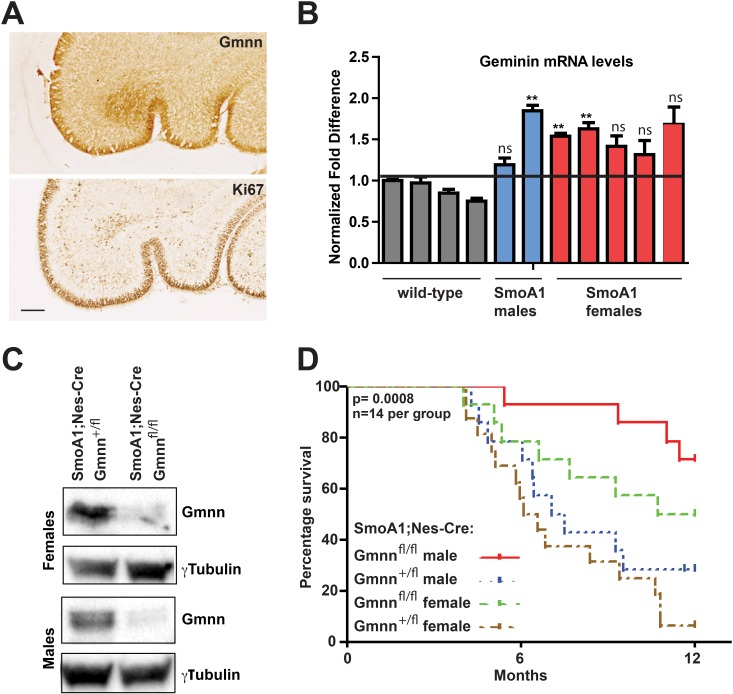
Geminin loss of function enhances survival in the SmoA1 medulloblastoma model (**A**) In the cerebellum of P5 mice, Gmnn is expressed in the EGL in a pattern like Ki67, which marks proliferative cells. Scale bar=100μm. (**B**) *Gmnn* mRNA levels were defined by qRTPCR analysis in P6 GNP preparations from individual wild-type (n=4) versus SmoA1 transgenic males (n=2) and females (n=5), and is expressed as a normalized fold difference. ^**^p<0.01 was defined by two-tailed student's t-test. ns=not significant. (**C**) Gmnn protein levels in SmoA1; Nes-Cre; Gmnn^+/fl^ versus Gmnn^fl/fl^ cerebella at P14, defined by Gmnn immunoblotting with γTubulin as a loading control. (**D**) Kaplan-Meier survival plot for cohorts of mice hemizygous for both the Nestin-Cre and SmoA1 transgenes and either heterozygous or homozygous for the floxed Gem allele. Animals were euthanized when they exhibited neurologic impairment or morbidity or at the end of the study and presence or absence of tumors was confirmed.

In the mouse, *Gmnn* was previously shown to be expressed in proliferating embryonic neural progenitors and adult neural stem cells, with downregulation of *Gmnn* expression accompanying the transition from progenitor to post-mitotic neuron [29, 51]. We examined its expression in the developing cerebellum, and found that *Gmnn* is highly expressed by GNPs in the EGL of the murine neonatal cerebellum at postnatal day 5 (P5), with expression overlapping Ki67, which marks all proliferating cells (Figure 2A). As conditional loss of Gmnn in the forming neural plate from ∼E8.0 in the mouse resulted in neural tube defects [29], we assessed later neurodevelopmental requirements here by performing Nestin-Cre-mediated conditional *Gmnn* excision. B6.Cg-Tg(Nes-Cre)1Kln/J (Jax 003771; hereafter Nes-Cre) mice express Cre recombinase under control of the rat nestin promoter and enhancer, predominantly in neural stem and progenitor cells of the central and peripheral nervous system (CNS/PNS) from embryonic day 10.5. Using this line, Gmnn protein levels in the CNS were previously shown to be strongly reduced by E14.5 [52]. Congruent with prior work [52], Nes-Cre; Gmnnfl/fl mice developed into normal, fertile adults. As described in detail below (Supplementary Figure 10), we further subjected these animals to a battery of behavioral assays and found that they exhibited no motor, cognitive, or other detectable behavioral abnormalities, which was consistent with prior work demonstrating a lack of neural stem cell or neurogenesis defects in this model [52]. Therefore, we used this model to test whether Gmnn deficiency could modify medulloblastoma formation or progression, by combining Nes-Cre mediated Gmnn excision with the SmoA1 model of murine medulloblastoma. This mouse model of the human SHH medulloblastoma subtype expresses an activated form of Smoothened (SmoA1) specifically in GNPs [53], under control of the NeuroD2 promoter (ND2) [54]. These mice exhibit excessive GNP proliferation and 48% of the animals develop medulloblastoma, with a median of 6 months of age [53].

We initially compared *Gmnn* expression levels in GNPs prepared from SmoA1 versus wildtype cerebellum at P6: *Gmnn* levels were elevated in SmoA1 transgenic animals (both males and females), compared to wild-type animals (Figure 2B). Cohorts of P14 male and female adults were then generated, all of which carried one copy of the Nes-Cre and SmoA1 transgenes and were either heterozygous or homozygous for the floxed *Gmnn* allele. Western blotting for Gmnn levels in cerebellar tissues from these SmoA1; Nes-Cre; Gmnn^fl/fl^ animals revealed complete loss of Gmnn protein by comparison to their SmoA1; Nes-Cre; Gmnn^+/fl^ littermates, which retained Gmnn expression (Figure 2C). We tracked cohorts of animals with these genotypes (n=14 animals in each group; including males and females) for one year. Adult SmoA1 mice that developed clinical symptoms of medulloblastoma (enlarged posterior fossa, tilted head, hunched posture, ataxia) were euthanized and histological sectioning of the brain was performed to confirm the presence of a tumor.

As seen previously for the SmoA1 medulloblastoma model [53], ∼50% of both males and females with a copy of wild-type *Gmnn* developed tumors and did not survive beyond 6 months. By contrast, animals homozygous for the *Gmnn* floxed allele (SmoA1;Nes-Cre; Gmnn^fl/fl^) had a substantial survival advantage, relative to SmoA1;Nes-Cre;Gmnn^+/fl^ animals that retained wild type Gmnn function (Figure 2D). This effect was significant for both males and females (Supplementary Figure 2), but was more pronounced for males, with ∼80% of the SmoA1; Nes-Cre; Gmnn^fl/fl^ animals surviving through the end of the study. Upon histological sectioning, all symptomatic animals exhibited large tumors that were usually detected bilaterally in parasagital sections through the brain, while very few animals that were asymptomatic through the end of the study exhibited a visible tumor mass (Supplementary Figure 3). We examined tumor size and histology by hematoxylin-eosin staining and also analyzed a subset of the tumors by immunofluorescence for Ki-67 and NeuN, marking proliferating and differentiated cells in the tumor, respectively (Supplementary Figure 3). All of these characteristics appeared similar in tumors with either the SmoA1;Nes-Cre;Gmnn^+/fl^or SmoA1;Gmnn^fl/fl^ genotypes. As tumors were processed for histology as animals in the cohort became symptomatic, these data did not reveal whether the rate of tumor growth was altered by Gmnn deficiency. However, the size, histology, and proliferative index of tumors that were analyzed did not appear to differ by genotype, despite the survival advantage conferred by Gmnn deficiency.

### Geminin deficiency reduces the persistence of preneoplastic cells in SmoA1 mice

The work above demonstrated that Gmnn deficiency could enhance survival in a model of medulloblastoma driven by constitutively active Shh signaling. Since both Gmnn-expressing and Gmnn-deficient cells could contribute to tumors, we examined whether the survival advantage conferred by Gmnn deficiency might relate to an altered propensity of preneoplastic cells to form tumors. In mouse models with constitutive Shh pathway activation, medulloblastoma tumorigenesis shows several stages of progression, with clusters of preneoplastic GNPs (pGNPs) that actively express Shh target genes such as cyclin D1 remaining on the surface of the mature cerebellum. These cells can be considered a preneoplastic intermediate, some of which develop into larger lesions and then tumors [55-58]. In SmoA1 transgenic mice, the tumorigenic effect of SmoA1 is balanced by tumor suppressive mechanisms, such that while all mice develop pGNPs, not all develop tumors. Control of proliferation, differentiation, and apoptosis all limit the growth of pGNPs [58, 59]. During the latent period that precedes tumor detection in multiple medulloblastoma mouse models, pGNPs acquire additional oncogenic changes that mediate progression from the precursor state to a fully tumorigenic state. Therefore, we assessed whether Gmnn deficiency could alter pGNP proliferation, differentiation, or survival during the postnatal period.

**Figure 3:**
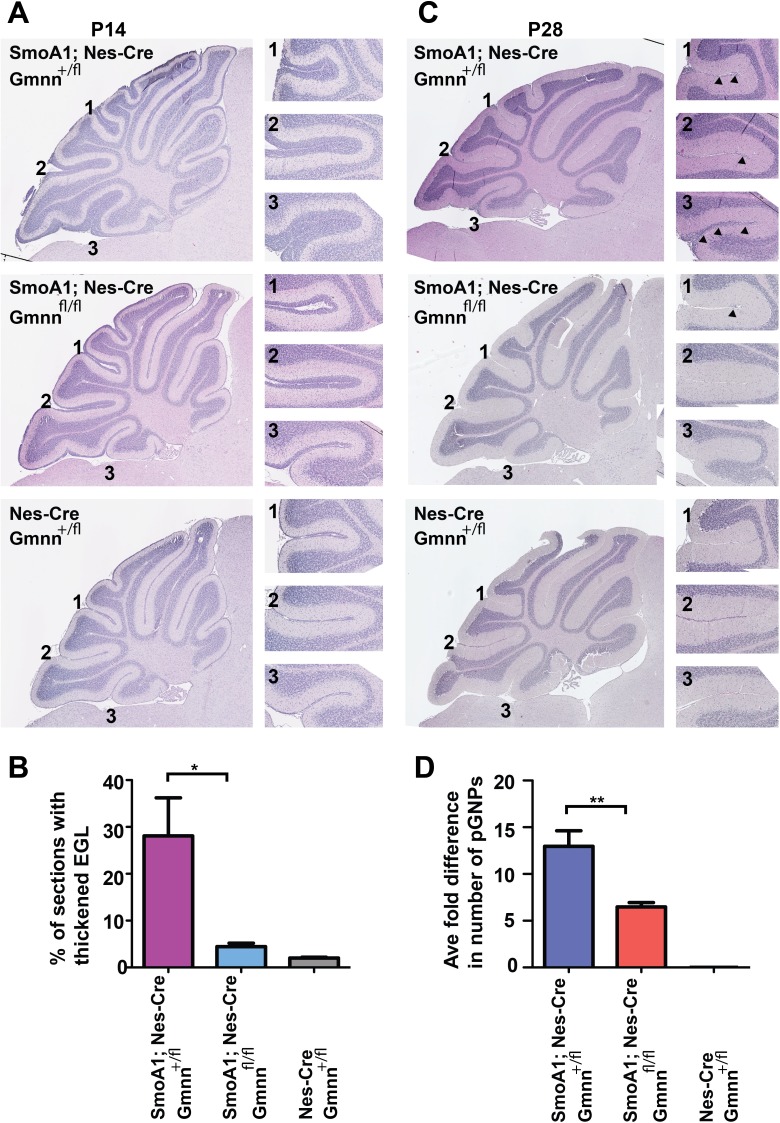
Geminin loss reduces numbers of persisting preneoplastic GNPs in SmoA1 transgenic mice (**A**) Thickness of the EGL layer in P14 cerebella hemizygous for the Nes-Cre and SmoA1 transgenes and heterozygous or homozygous for the *Gmnn* floxed allele. Control comparison to animals not carrying the SmoA1 transgene is also shown. Matched cerebellar sections with insets corresponding to labelled regions at right. (**B**) Quantitation for A, n=3 animals for each genotype, with matched 5μm sections analyzed for each animal (see Methods) and the percentage of sections with >2-3 persisting EGL cell layers scored as thickened for 3 animals for each of the three genotypes. (**C**) At P28, cerebella of the same genotypes as in A are shown, with pGNPs persisting at the cerebellar surface staining darkly in the H&E stained brain sections (marked by arrowheads). (**D**) Averaged counts/section were from 4 matched serial sections per P28 brain. Number of animals per genotype analyzed: n=11 (SmoA1; Nes-Cre; Gmnn^+/fl^), n=10 (SmoA1; Nes-Cre; Gmnn^fl/fl^), n=6 (Nes-Cre; Gmnn^+/fl^). ^*^<0.05, ^**^<0.01 were defined by two-tailed student's t-test.

To assess whether Gmnn deficiency modified the growth or survival of preneoplastic GNPs, we initially analyzed early hyperplastic lesions in the SmoA1 medulloblastoma model. During normal development, the P14-P16 EGL is comprised of 2-3 GNP cell layers, as most GNPs have completed their migration into the IGL (for example, in Nes-Cre; Gmnn^+/fl^ animals lacking the SmoA1 transgene, Figure 3A). At this time, mice with a single copy of the SmoA1 transgene have thickened EGL regions, expanded as nodular formations within the cerebellar lobules. At P14, heterozygous mice that retained Gmnn activity (SmoA1; Nes-Cre; Gmnn^+/fl^) displayed thicker EGL regions than animals homozygous for the Gmnn floxed allele (SmoA1; Nes-Cre; Gmnn^fl/fl^) (Figure 3A-3B). This phenotype was also seen in cerebellar sections from SmoA1 transgenic animals at P16, with increased EGL thickness apparent in SmoA1; Nes-Cre; Gmnn^+/fl^ animals by comparison with SmoA1; Nes-Cre; Gmnn^fl/fl^ animals (Supplementary Figure 4). These data suggest that, relative to heterozygous animals expressing Gmnn, loss of Gmnn results in fewer SmoA1 expressing GNPs with preneoplastic potential remaining at the cerebellar surface.

By four postnatal weeks (P28), the EGL has largely disappeared (Figure 3C, Nes-Cre; Gmnn^+/fl^ animal lacking the SmoA1 transgene). However, in mice carrying the SmoA1 transgene at this stage, foci of ectopic proliferative preneoplastic GNPs (pGNPs) are still present on the external cerebellar surface. These may be classified as focal hyperplasias (<30 pGNPs) or diffuse hyperplasias (>5000 cells). As many of these cells do not develop into tumors, medulloblastoma incidence is influenced both by the frequency with which GNPs become preneoplastic in early postnatal life and with the survival, persistence, and continued proliferation of these cells to form tumors. We therefore generated sex-matched littermates that carried one copy of the SmoA1 and Nestin-Cre transgenes and were also either heterozygous or homozygous for the floxed Gmnn allele, comparing cerebellar sections from these animals at P28. Animals homozygous for Gmnn loss (SmoA1; Nes-Cre; Gmnn^fl/fl^) had six-fold fewer preneoplastic cells than SmoA1; Nes-Cre; Gmnn^+/fl^ animals retaining wild-type Gmnn (Figure 3C-3D, pGNP cell counts for individual animals are in Supplementary Figure 5). In addition, we found that some P28 SmoA1; Nes-Cre; Gmnn^+/fl^ animals had severe cerebellar dysplasia, while we never observed this phenotype in SmoA1; Nes-Cre; Gmnn^fl/fl^ animals (Supplementary Figure 6). Together, these data suggest that Gmnn deficiency alters the proliferation, differentiation, and/or survival of pGNPs. Therefore, we next assessed whether alteration of these properties could account for the significant decrease in pGNPs under conditions of Gmnn deficiency in the SmoA1 transgenic model.

### Geminin deficiency reduces the persistence of preneoplastic granule neuron precursor cells in the SmoA1 model by activation of a DNA damage response and apoptosis

In our work above, we demonstrated that Nes-Cre-driven Gmnn deficiency enhanced survival in the SmoA1 medulloblastoma mouse model. Likewise, at P28, SmoA1; Nes-Cre; Gmnn^fl/fl^ cerebella exhibited significantly fewer pGNPs than were seen in cerebella of SmoA1; Nes-Cre; Gmnn^+/fl^ animals. These data suggested that Gmnn deficiency compromised the persistence of pGNPs, which could account for decreased rates of tumor formation in these animals. This reduction in numbers of persisting pGNPs could result from enhanced differentiation, diminished proliferation, or altered survival of pGNPs.

**Figure 4:**
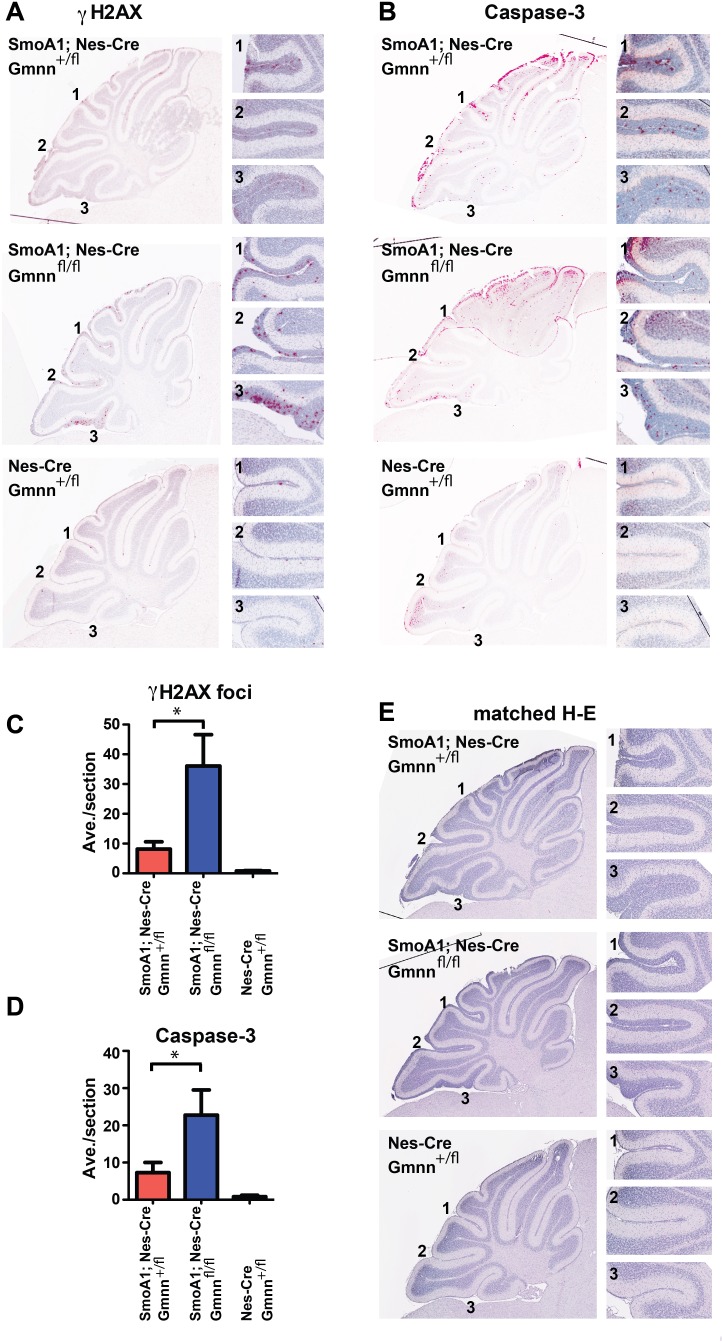
Geminin loss increases pGNPs expressing markers of the DNA damage response and apoptosis Immunohistochemistry for γH2AX and cleaved (active) Caspase3 on P14 cerebellar sections from animals hemizygous for the Nes-Cre and SmoA1 transgenes and either heterozygous or homozygous for the Gmnn floxed allele. Matched representative sections are shown for each genotype in (**A-B**), with matched hematoxylin-eosin stained sections in E. (**C-D**) Immunopositive pGNPs were quantified and represented as average counts/section with ^*^<0.05 defined by two-tailed student's t-test.

To address which of these mechanisms could contribute to the reduced numbers of pGNPs observed in the SmoA1; Nes-Cre; Gmnn^fl/fl^ animals, we immunostained cerebella from P14 SmoA1; Nes-Cre; Gmnn^+/fl^ and SmoA1; Nes-Cre; Gmnn^fl/fl^ littermates with antibodies against γH2AX (induction of DNA damage response), cleaved (active) Caspase-3 (apoptotic marker), phosphorylated histone H3 (pH3; mitotic cell marker), p27^kip1^ (cell cycle exit accompanying GNP differentiation), and CyclinD1 (proliferative cell marker). We observed a significant increase in the fraction of pGNPs expressing both γH2AX (Figure 4A, 4C) and active Caspase-3 (Figure 4B, 4D) in SmoA1; Nes-Cre; Gmnn^fl/fl^ cerebella, by comparison with littermates that retained Gmnn activity (SmoA1; Nes-Cre; Gmnn^+/fl^). This result suggests that Gmnn loss results in increased accumulation of DNA damage and apoptotic cell death of pGNPs. An adjacent matched section for each genotype was also stained with hematoxylin and eosin, confirming the differences in overall thickness of the EGL in the sections examined (Figure 4E). By contrast, there did not appear to be significant differences in the fraction of pGNPs that were immunopositive for Cyclin D1, p27^kip1^, or pH3 in the SmoA1; Nes-Cre; Gmnn^+/fl^ versus SmoA1; Nes-Cre; Gmnn^fl/fl^ backgrounds (Supplementary Figure 7), suggesting that Gmnn deficient pGNPs could still proliferate and undergo cell cycle exit and differentiation. These results suggest that Gmnn loss sensitizes pGNPs to accumulation of DNA damage and apoptosis, which could account for the reduced numbers of persisting pGNPs and the enhanced survival observed in SmoA1; Nes-Cre; Gmnn^fl/fl^ animals.

### Geminin knockdown induces cell cycle arrest of human medulloblastoma cells

**Figure 5:**
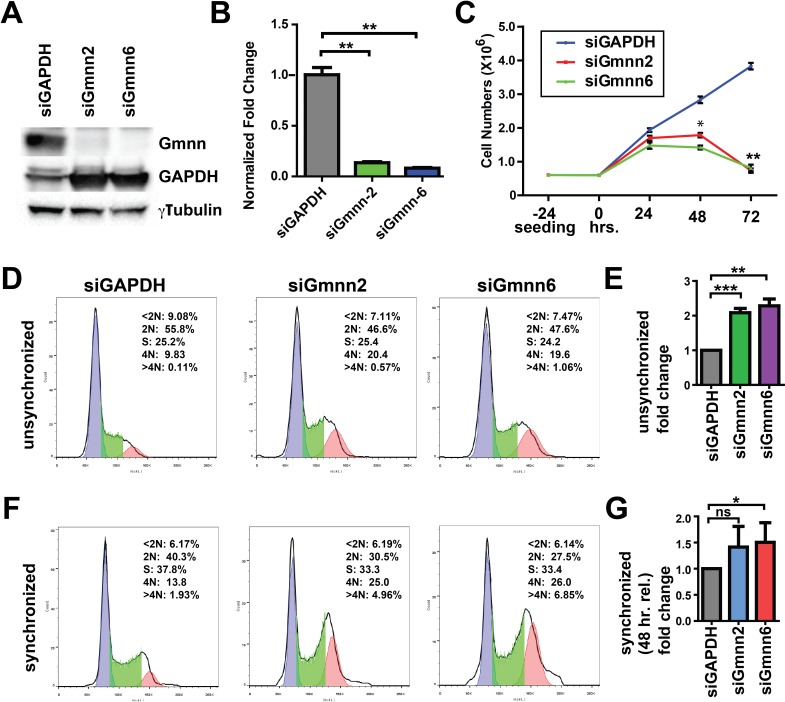
Geminin knockdown causes DNA re-replication and G2/M cell cycle arrest in human medulloblastoma cells **(A)**. Gmnn siRNA knockdown in Daoy cells was performed using 25 nM of Gmnn siRNAs #2 or #6 (see Methods) and levels of knockdown were assessed after 72hrs by Western blotting (A) and by qRTPCR **(B)**, revealing ∼90% knockdown achieved with each siRNA. **(C)** Growth curve: 6×10^5^ cells were seeded and siRNA transfection was performed 24 hrs later (time 0). Numbers of cells were counted 24, 48, and 72 hr. post-transfection. (**D-E**) 72 hrs. after Gmnn siRNA knockdown, cells were stained with propidium iodide and 1×10^6^ cells for each condition were analyzed for DNA content using FACS. Representative FACS plots are shown in D. In E, the fraction of cells with 4N DNA content following Gmnn siRNA knockdown is represented as a fold ratio relative to the GAPDH siRNA control. An average value obtained from four independent biological replicates is shown. (**F-G**) Daoy cells were synchronized with hydroxyurea as described (Methods) and effectiveness of synchronization (arrest of cells in G1/S) is shown in Figure S9. Percentages of cells in G2/M were determined by FACS analysis and quantified as described in D-E, for 3 independent biological replicates. p-values: ^*^<0.05, ^**^<0.01,^***^<0.001 were defined by two-tailed student's t-test.

SmoA1 pGNPs and tumor cells from the mouse model are not amenable to long-term culture *ex vivo* (data not shown). Therefore, to further assess whether Gmnn deficiency could affect medulloblastoma cell proliferation, growth, and/or survival, we also manipulated Gmnn levels in Daoy cells, a widely used human medulloblastoma cell line that mimics some characteristics of the Shh medulloblastoma subtype [60-62]. We performed a dose response curve to identify the lowest concentration of small interfering RNAs (siRNAs) that significantly reduced Gmnn levels in Daoy cells (Supplementary Figure 8). We validated two independent siRNAs directed against human Gmnn, each of which induced 85-90% reduction in Gmnn protein levels, with siGmnn#6 consistently reducing Gmnn levels even more strongly than siGmnn#2 (Figure 5A-5B). Acute Gmnn knockdown with both siRNAs significantly impaired Daoy cell growth *in vitro*, resulting in a plateau and subsequent decline in cell numbers (Figure 5C).

**Figure 6:**
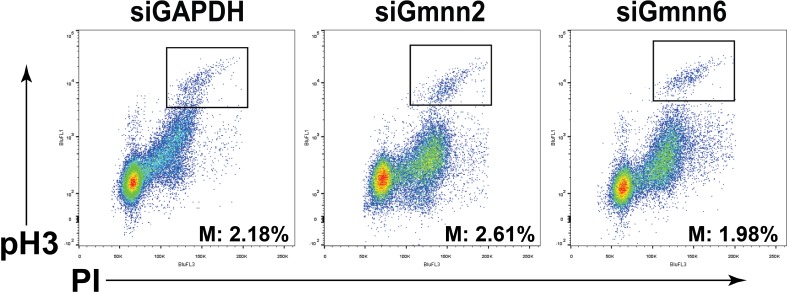
Geminin knockdown results in cell cycle arrest in G2 rather than M phase Representative FACS plots of cells analyzed for DNA content (PI: x-axis) and by immunocytochemistry for pH3 (y-axis). Similar percentages of highly pH3 immunopositive cells (M-phase cells) were seen in all three knockdown conditions.

We conducted cell cycle analysis in Daoy cells 72 hours after Gmnn or control (GAPDH) siRNA knockdown, without prior synchronization of the cell population. Gmnn knockdown resulted in a significant accumulation of cells with 4N DNA content, e.g. in the G2/M phases of the cell cycle (Figure 5D-5E). To better quantify the increased numbers of 4N (G2/M phase) cells following Gmnn knockdown, we synchronized the cell population in G1/S by hydroxyurea treatment (treatment conditions defined in Supplementary Figure 9). Gmnn knockdown likewise induced accumulation of these synchronized Daoy cells in G2/M, with pronounced effects seen in synchronized cell populations 48 hrs. after hydroxyurea release, corresponding to 72 hrs. of Gmnn knockdown (Figure 5F-5G). We next assessed whether Gmnn knockdown caused Daoy cells to arrest in the G2 or M phase of cell cycle, by combining FACS for a mitotic marker (phosphorylated histone H3; pH3) with propidium iodide analysis for cellular DNA content. No reproducible differences in numbers of M-phase cells were detected in the Gmnn siRNA condition, by comparison with the GAPDH siRNA control (Figure 6). These results suggest that Gmnn knockdown causes significant accumulation of cells in the G2 phase rather than M phase of the cell cycle.

### Geminin depletion induces activation of G2 checkpoint and DNA damage response pathways and apoptosis in human medulloblastoma cells

**Figure 7:**
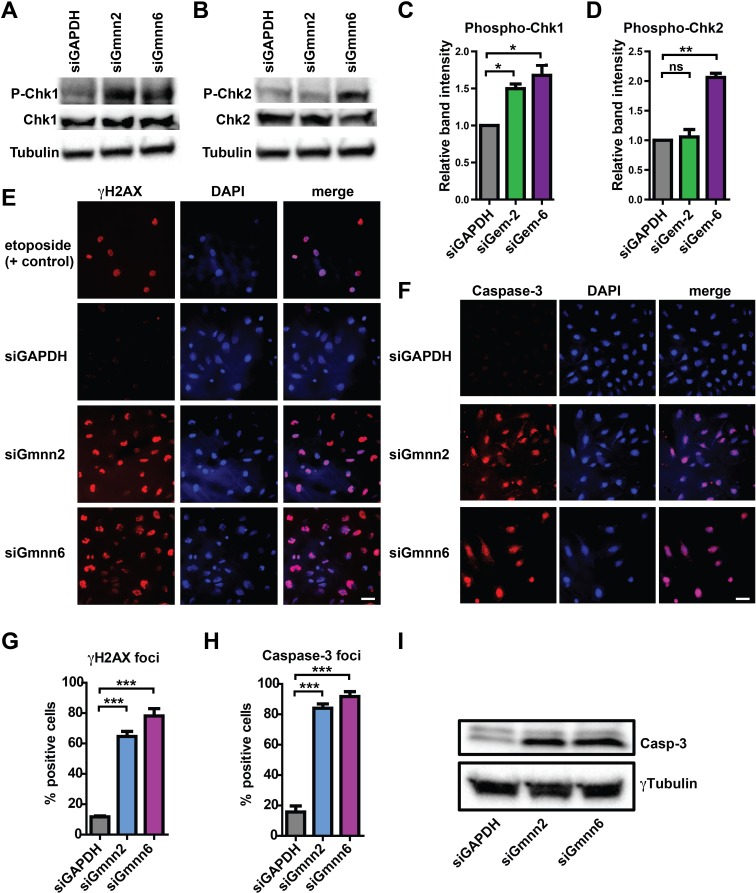
Geminin depletion induces G2 checkpoint activation, DNA damage response, and apoptosis in human medulloblastoma cells (**A-B**) siRNA knockdown was performed on unsynchronized Daoy cells as above. Representative westerns for phosphorylated-Chk1 and Chk2 are shown relative to total Chk1/2 and to the γTubulin loading control. (**C-D**) Quantitation of changes in phosphorylated Chk1/2 was performed by quantification of band intensity and is represented as the average of two experiments. **(E-F)** Immunocytochemistry for γH2AX and activated Caspase-3 was performed on siRNA transfected cells. Representative images are shown, with etoposide used as a positive control to induce DNA damage in E. Scale bars=50μm. (**G-H**) Quantification of data from E-F was obtained by scoring >250 cells per sample type per experiment for three independent experiments. **(I)** Western blotting for cleaved caspase was assessed in the siRNA transfected cells. p-values: ^*^<0.05, ^**^<0.01, ^***^<0.001 were defined by two-tailed student's t-test. ns=not significant.

Gmnn deficient Daoy cells accumulated in the G2 phase of the cell cycle. This could potentially occur subsequent to DNA damage incurred during S phase, such as DNA re-replication by unimpeded Cdt1 activity, and the resulting replication stress would be expected to activate DNA damage checkpoint pathways. Therefore, to assess checkpoint pathway activation, we compared levels of phosphorylated, activated Chk1 and Chk2 checkpoint kinases in control versus Gmnn knockdown Daoy cells. Both Gmnn siRNAs strongly induced activation of Chk1, while the more effective knockdown construct (siGmnn#6) also induced strong activation of Chk2 (Figure 7A-7D).

As recruitment of γH2AX to chromatin is an early indicator of activation of DNA damage repair processes, we performed immunostaining for γH2AX to test whether Gmnn deficiency triggered a DNA damage response. Etoposide treated Daoy cells were used as a positive control for DNA damage and these cells stained positive for γH2AX (Figure 7E). While γH2AX immunopositive cells were not seen in control siRNA transfected cells, Gmnn depletion with both siRNAs resulted in formation of γH2AX -positive foci in >75-80% of cells (Figure 7E, 7G). As failure to repair DNA damage may trigger cell death by apoptosis, we also assessed the status of cleaved Caspase-3, a marker for apoptosis, in control and Gmnn knockdown cells. By comparison with control cells, knockdown of Gmnn significantly increased levels of cleaved Caspase-3, as assessed by both immunostaining and Western blotting analyses (Figure 7F, 7H-7I).

### Interference with Geminin activity acts synergistically with the topoisomerase inhibitor etoposide

Standard treatment for medulloblastoma includes maximal safe tumor resection, followed by craniospinal irradiation and multi-agent chemotherapy [39]. The topoisomerase inhibitor etoposide (VP-16) has been used as a component of combinatorial chemotherapy for newly diagnosed and recurrent medulloblastoma [63-65]. Interestingly, in addition to interacting with Cdt1, Gmnn can interact with Topoisomerase IIα (Topo IIα), a target for etoposide, which resolves chromosomal catenanes formed during DNA replication [12, 66]. Gmnn was reported to both promote recruitment of Topo IIα to chromatin during replication and to stimulate its release from chromosomes. Accordingly, in some cell contexts, Gmnn over-expression triggers premature Topo IIα release from chromosomes, increasing cellular resistance to the Topo IIα poisons etoposide and doxorubicin, which act by trapping the chromosomally bound transient reaction intermediate [12, 66].

**Figure 8:**
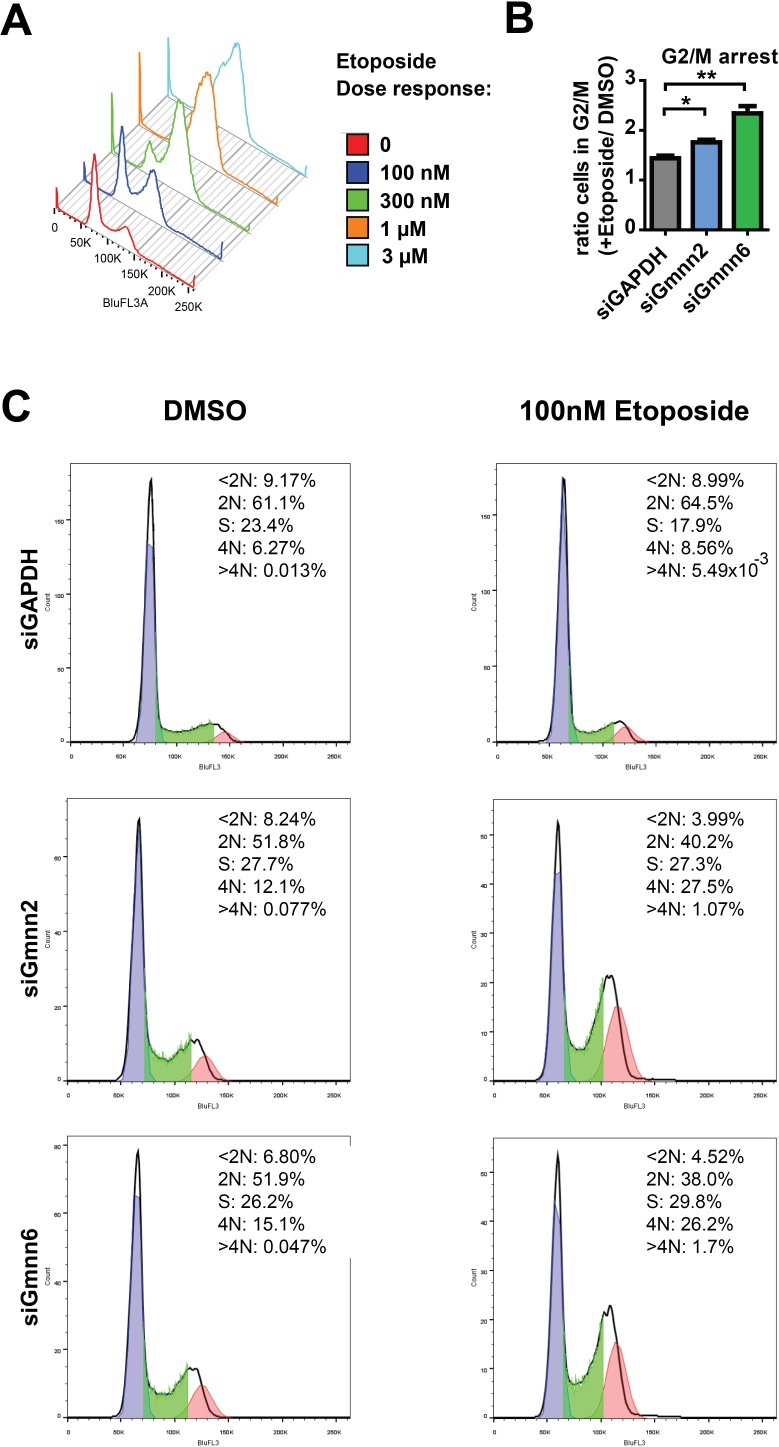
Interference with Geminin activity acts synergistically with the topoisomerase inhibitor etoposide (**A**) Etoposide dose response curve was generated by treating Daoy cells with increasing concentrations of etoposide and evaluation of DNA content by propidium iodide staining and FACS analysis. 100 nM was selected as a dose for assessing interactions with Gmnn knockdown in subsequent experiments. (**B-C**) PI FACS analysis as described previously was performed on Gmnn siRNA knockdown cells with addition of DMSO vehicle or 100 nM etoposide. (**B**) Quantification of three independent experiments. Ratio of G2/M (4N) cells in etoposide, compared with the DMSO control, is shown for each knockdown condition. (**C**) One representative experiment (of three independent experiments) is shown. p-values: ^*^<0.05, ^**^<0.01 were defined by two-tailed student's t-test.

Since Gmnn can interact with Topo IIα, and Topo IIα inhibitors like etoposide are used to treat medulloblastoma, we additionally investigated whether Gmnn inhibition could potentiate etoposide efficacy. We began by performing a dose-response for etoposide treatment of Daoy cells, to identify a dose that caused a modest accumulation of cells in the G2 phase (Figure 8A). We used this dose (100nM) of etoposide in combination with control or Gmnn knockdown and found that Gmnn inhibition using either siRNA acts additively with etoposide to increase the G2 accumulation of Daoy cells (Figure 8B-8C). Taken together, these data suggest that inhibition of Gmnn can induce cell cycle arrest, DNA damage checkpoint activation, and apoptosis of medulloblastoma cells, and could potentially sensitize tumor cells to combination chemotherapies involving etoposide.

### Nestin-Cre-mediated Geminin excision in the nervous system does not alter motor, cognitive, or other behavioral functions

Since medulloblastoma develops during childhood, selective therapies should target cancer cells without affectingnormalneurodevelopmentorneurological function. Therefore, to determine whether Gmnn inhibition represented a plausible approach for selective targeting of cancer cells in medulloblastoma, we thoroughly examined effects of Gmnn loss on neurological function in the Nes-Cre conditional knockout model. Using a range of behavioral assays, we assessed age-matched male and female cohorts of adults carrying one copy of the Nes-Cre transgene that were either wild-type for Gmnn or homozygous for the floxed Gmnn allele. We first assessed possible impairment of learning and memory and simple motor and sensorimotor functions, which might confound performance in cognitive tests. This involved quantifying performance during a one hour locomotor activity and exploratory behavior test, a battery of sensorimotor measures, and the Morris water navigation task. We also conducted a series of additional tests at a slightly older age to evaluate other behavioral parameters. These included the elevated plus maze, acoustic startle/pre-pulse inhibition of startle, hole board exploration/olfactory preference, and social approach, in that order. None of these tests revealed a distinct behavioral phenotype resulting from Nes-Cre-mediated conditional loss of Gmnn function (Supplementary Figure 10 and Supplemental Results and Methods). Our behavioral testing efforts do not qualify as a definitive evaluation of all possible untoward effects of conditional loss of Gmnn function. However, our data suggest that therapeutic interventions involving targeting of Gmnn activity in the developing nervous system are unlikely to have major deleterious effects on neurodevelopment or neurological function.

## DISCUSSION

### Geminin deficiency suppresses medulloblastoma tumorigenesis

The Shh-driven subtype accounts for about 30% of all medulloblastomas [44, 67], with GNPs serving asthe cell of origin [68, 69]. During cerebellar development in the mouse, GNP proliferation in the EGL is maximal at about one week after birth, while by three weeks the GNPs have differentiated and migrated into the IGL. In the SmoA1 model at P14-P16, the EGL remains thickened with clusters of GNPs with preneoplastic potential remaining at the cerebellar surface. However, Gmnn deficiency partially suppressed these effects. These effects were also seen at four postnatal weeks, when Gmnn loss reduced the persistence of pGNPs at the cerebellar surface by six-fold in the SmoA1 model. These findings correspond with altered frequencies of tumorigenesis in cohorts of SmoA1 transgenic animals with versus without Gmnn loss, with Gmnn loss conferring a significant survival advantage. These data suggest that Gmnn deficiency may modify pGNP tumorigenic potential from an early developmental timepoint, diminishing pGNP persistence at the cerebellar surface and reducing the rate at which medulloblastoma tumors form under conditions of constitutively active Shh signaling.

As modulating pathways that affect pGNP proliferation, differentiation, or survival can all affect the frequency of tumor formation [49, 70], we assessed which processes were affected by Gmnn deficiency in the SmoA1 model. We found no significant difference in the fraction of pGNPs expressing cyclin D1, the mitotic marker pH3, or p27Kip1 in cerebella of SmoA1 retaining versus with loss of Gmnn expression. These data suggest that loss of Gmnn did not grossly alter the ability of pGNPs to proliferate, exit the cell cycle, or differentiate. By contrast, Gmnn loss in the SmoA1 model significantly increased relative numbers of pGNPs immunopositive for γH2AX, which marks cells with DNA damage, and cleaved Caspase-3, which labels cells undergoing apoptosis. These data suggest that Gmnn deficiency can trigger a DNA damage response in pGNPs in the SmoA1 model from early stages, elevating rates of pGNP apoptosis. This may account for the reduced numbers of pGNPs persisting at the cerebellar surface at later postnatal stages and for the survival advantage later observed in these animals.

### Geminin is required to maintain the fidelity of DNA replication in medulloblastoma

The pre-RC protein Cdt1 is essential for loading DNA helicases onto origins of DNA replication. Cdt1 inactivation prevents genome re-replication within a cell cycle and occurs through multiple mechanisms [4-9]. During S-phase, cyclinA:CDK2 complexes phosphorylate Cdt1, targeting it for SCF^Skp2^–dependent ubiquitination, while CDR^Ddb1^ also ubiquitinates Cdt1, both of which promote Cdt1 proteolysis. Gmnn's ability to specifically bind to Cdt1 during S/G2 phases of the cell cycle constitutes a third system for controlling Cdt1 activity, both preventing Cdt1 from reinitiating DNA replication and also protecting Cdt1 from proteolytic degradation [1, 2, 4, 7, 9].

Since Gmnn inhibits Cdt1, Gmnn depletion can result in genome re-replication within a cell cycle, through increased Cdt1 activity. However, prior work had suggested that cells derived from normal tissues and cancer cells may exhibit differential dependence upon Gmnn to prevent DNA re-replication [10]. In Zhu *et al*., siRNA knockdown of Gmnn caused re-replication and triggered apoptosis in a wide variety of cancer cell lines, while inducing similar effects in non-cancer cell lines required combined suppression of Gmnn and Cyclin A [10]. These data suggest that Gmnn may be the primary regulator of Cdt1 inhibition in cancer cells, while additional safeguards are sufficient to constrain DNA re-replication to maintain genome fidelity in many non-cancer cell contexts. Preferential sensitivity of some cancer cells to Gmnn depletion may involve their overexpression of pre-RC proteins including Cdt1 and Cdc6, which stimulate pre-RC complex re-assembly and re-firing of origins of replication within the same cell cycle [11]. Gmnn expression is elevated in many tumor cells, and may constrain this additional pre-RC complex activity, such that altering this balance by Gmnn depletion would preferentially trigger DNA damage due to re-replication and checkpoint arrest in cancer cells. Interestingly, here we found that Gmnn levels in human medulloblastomas did not correlate with tumor subtype, histology, or other criteria, but did correlate with elevation of cell cycle related genes, including pre-RC proteins (MCM6, ORC1, and ORC6). This would be compatible with a need for elevated Gmnn to balance increased pre-RC expression, to maintain genome integrity and facilitate tumor growth.

Gmnn inhibition elicits distinct, cell type-specific responses. In some cells, Gmnn inhibition results in extensive genome re-replication, with G2 checkpoint arrest triggered by production of aneuploid cells with >4N DNA content. However, other cell types (for example, immortalized human mammary epithelial cells) undergo mitotic rather than G2 arrest upon Gmnn silencing [66, 71]. In these cells, Gmnn loss does not cause S phase progression defects or genome over-replication, but rather causes mitotic arrest, inability to exit mitosis, and the formation of unresolved chromosomal bridges. We found that inhibition of Gmnn in human Daoy medulloblastoma tumor cells caused accumulation of cells in G2 without significant changes in numbers of M-phase cells. However, unlike the cancer cell lines characterized by Zhu *et al*., in which 28-44% of cells exhibited extensive DNA re-replication [10], Gmnn depletion in Daoy cells resulted in a much more modest fraction of >4N cells. However, 70-80% of these cells accumulated γH2AX-positive DNA damage foci and underwent spontaneous apoptosis, measured by cleaved Caspase-3 immunopositivity.

Blow and colleagues have suggested that levels of re-replication resulting from Gmnn depletion may be influenced by the length of G2 arrest, with prolonged G2 checkpoint arrest enabling Cdt1 accumulation, which promotes additional genome re-replication and greater DNA damage [72]. This may constitute a protective response, making re-replication into an all-or-nothing phenomenon to prevent accumulation of cells with low levels of re-replication [72]. Modest induction of re-replication subsequent to either Gmnn deficiency or Cdt1 overexpression is sufficient to induce G2 checkpoint activation [72, 73]. Inappropriate re-licensing can also result in other forms of DNA damage, including head-to-tail replication fork collision and the tendency of re-replicating forks to collapse [74, 75]. Therefore, even minor induction of re-replication or other types of replication stress (for example, those related to altered Topo IIα decatenation activity) could also damage the genome in S phase in a manner that may not be apparent as a significant fraction of G2/M cells with >4N DNA content. The ability of minor amounts of replication stress to trigger a DNA damage response and subsequent apoptosis could account for the results seen here *in vivo*. In the SmoA1 model, Gmnn deficiency strongly induced γH2AX and cleaved Caspase-3 immunopositivity in pGNPs. By P28, these Gmnn deficient animals had six-fold fewer pGNPs at the cerebellar surface, likely contributing to the enhanced survival of the Gmnn-deficient cohort. However, the SmoA1 tumors that did form in Gmnn-deficient animals had similar fractions of proliferative cells as those without Gmnn deficiency, while the fraction of pGNPs expressing proliferative cell markers was not grossly altered by Gmnn deficiency. These findings suggest that Gmnn-deficient preneoplastic and cancer cells may be sensitized, potentially by incurring DNA damage, to undergo apoptosis even under conditions where they do not exhibit cell cycle arrest or grossly altered proliferative capacity.

Both in mouse *in vivo* and human *in vitro* models of medulloblastoma, Gmnn deficiency triggered a DNA damage response and apoptosis. In human medulloblastoma cells, the DNA damage response was accompanied by activation of both ATR/Chk1, which is activated in response to DNA damage other than double strand breaks, including replication stress, and ATM/Chk2, which is activated predominantly in response to double strand breaks [76, 77]. Interestingly, Gmnn knockdown using the two siRNAs yielded slightly different results, with siGmnn-2 only activating pChk1 while siGmnn-6 activated both pChk1 and pChk2. The siGmnn-6 construct yields higher knockdown of Gmnn compared to siGmnn-2, suggesting that the extent of Gmnn loss may affect the nature and extent of DNA damage that occurs upon Gmnn knockdown in medulloblastoma cells, with the siGmnn-2 construct predominantly triggering replication stress leading to ATR/Chk1 activation, while more effective knockdown with siGmnn-6 resulted both in higher levels of replicative stress and induction of double strand DNA breaks, activating both pChk1 and pChk2.

In some cancer cell contexts, effects of Gmnn inhibition have been shown to be independent of the mutational status of the tumor suppressor TP53, with Gmnn loss inducing accumulation of excess genomic DNA, G2/M checkpoint activation, and apoptosis in both TP53+ and TP53-cells [10, 78, 79]. We focused our *in vitro* studies here on the Daoy human medulloblastoma cell line. Daoy cells are the oldest established medulloblastoma cell line: they were first isolated in 1985 from a 4-year old boy [60] and have been utilized in more studies than any other medulloblastoma cell line [61], with molecular subtyping indicating that they mimic the SHH pathway activated subtype of medulloblastoma [62]. The Daoy cell line harbors a single point mutation of the tumor suppressor TP53, resulting in a substitution of phenylalanine for a cysteine at amino acid 242 and loss of functional p53 [80, 81]. By contrast, the SmoA1 mouse model used here has intact TP53 activity. TP53 mutations are only found in a fraction of human medulloblastomas of the Wnt (16%) and Shh (21%) subtypes, although they correlate with poor survival and treatment failures in Shh-driven medulloblastoma [38, 82, 83]. However, in both of our experimental models, loss of Gmnn resulted in a similar induction of the DNA damage response and apoptosis. This suggests that, as in other cancer cell contexts, Gmnn inhibition can elicit some effects regardless of TP53 status, such that Gmnn-directed therapeutics could potentially have similar efficacy in both TP53+ and TP53-forms of medulloblastoma.

### Geminin deficiency sensitizes medulloblastoma cells to etoposide treatment

Another mechanism by which Gmnn levels could influence genome integrity is through interactions with Topo IIα, which removes catenated intertwines between sister chromatids to separate replicated chromosomes. Gmnn-Topo IIα interaction may facilitate Topo IIα activity during termination of DNA replication [66]. In human mammary epithelial (HME) cells, physiologically normal Gmnn levels are needed to both facilitate Topo IIα recruitment to chromosomes, by promoting Topo IIα phosphorylation, and for recruitment of deSUMOylating enzymes to Topo IIα, to promote its release from chromatin. In that work, Gmnn over-expression promoted premature Topo IIα release from chromatin and increased the resistance of HME and breast cancer cells to Topo IIα inhibitors such as etoposide, which trap the transient Topo IIα reaction intermediate on chromatin to prevent DNA religation and promote cell death. These findings suggested that the high levels of Gmnn expression we observed in medulloblastoma could contribute to etoposide resistance, while inhibiting Gem might chemosensitize cells in multi-agent chemotherapy treatments involving etoposide. We found that Gmnn inhibition indeed enhanced the responsiveness of medulloblastoma cells to etoposide treatment, significantly increasing the fraction of cells that underwent G2 checkpoint arrest. Therefore, given the utilization of etoposidase and other Topo IIα inhibitors in current medulloblastoma chemotherapies, inhibition of Gmnn in this context might have the potential to enhance efficacy and/or re-sensitize resistant cells. We performed these experiments in the Daoy cell line, because it is amenable to *in vitro* culture, while we could not culture tumor cells from the SmoA1 mouse model *ex vivo*. However, the Daoy model has limitations as a model of medulloblastoma: it is hypertetraploid, with a modal chromosome number of 93-99, and carries a TP53 mutation, while most human medulloblastomas do not exhibit these features [60, 61, 80, 81]. Therefore, in future studies, it would be of interest to test whether Gmnn loss or inhibition could also enhance the efficacy of etoposide treatment in the context of *in vivo* models of medulloblastoma.

Since medulloblastoma is a childhood cancer that occurs during development and maturation of the nervous system, we also assessed whether Gmnn inhibition could be used to selectively target cancer cells without affecting nervous system development or neurological function. Prior work describing neurodevelopmental phenotypes of Nes-Cre-mediated Gmnn loss has yielded conflicting results. Schultz *et al*. described these animals as having normal neural stem cell function and normal neurogenesis [52], while Spella *et al*. suggested that Nes-Cre-driven Gmnn loss led to expansion of neural progenitors during ex vivo cortical progenitor culture [84]. One experimental variable that may underlie these conflicting findings is use of different Nes-Cre transgenic lines with distinct sites of integration, which may influence transgene expression. Our work here used (Nes-cre)1Kln, which was previously documented to result in significantly diminished Gmnn protein in CNS tissues by E14.5 [52]. Using this Nes-Cre driver, Gmnn loss did not result in detectable alterations of neurological function, which was assessed by conducting an extensive panel of behavioral and motor assays. Therefore, these data suggest that Gmnn requirements for neural development are restricted to early embryogenesis and support the potential utility of Gmnn directed therapeutics in selective targeting of cancer cells in medulloblastoma.

Efforts are underway to define small molecules that induce cancer-selective induction of re-replication, cell cycle arrest, and spontaneous apoptosis, by inhibiting the activity of Gmnn or of other molecular controls that prevent excessive DNA replication [12, 78]. Our work here demonstrates that, while Gmnn appears largely dispensable for normal neurodevelopment and normal neurological function after early embryogenesis, Gmnn inhibition can impair the growth of both preneoplastic and tumor cells in medulloblastoma, inducing them to undergo a DNA damage response and apoptosis. These effects were observed both in a mouse *in vivo* model of medulloblastoma and in human medulloblastoma cells, and resulted in increased survival in the mouse model, while Gmnn deficient medulloblastoma cells were also sensitized to treatment with the Topo IIα inhibitor etoposide. As anticancer agents targeting Gmnn become available, our work here suggests they may have utility in combination chemotherapies targeting medulloblastoma.

## MATERIALS AND METHODS

### Human tumor expression

Comparison of *Gmnn* expression in normal cerebellum versus in medulloblastoma tumors used the R2: MegaSampler (hgserver1.amc.nl/cgi-bin/r2/main.cgi). Data platform is u133p2 [HG-U133_Plus_2] Affymetrix Human Genome U133 Plus 2.0 *Array*, MAS5.0 with normalization applied by R2) 2log expression data was compared for normal cerebellum (sample accession: Roth; n=9; GSE3526) versus four sets of medulloblastoma samples (Gilbertson, n=76, GSE37418; Pfister; n=73, GSE49243; Delattre, n=57; Kool, n=62, GSE10327).

### Mouse strains and animal husbandry

Animal studies were conducted under protocols approved by the Washington University Institutional Animal Care and Use Committee. Gmnn conditional knockout mice (CSD24729) were purchasedfrom the Knockout Mouse Project (KOMP). This targeted Gmnn allele contains a splice acceptor-βgeo-polyA sequence flanked by FRT sites (permitting excision by FLPe recombinase) inserted into intron 3 and loxP sites flanking exon 4 (permitting excision of exon 4 by Cre recombinase). Prior to FLPe excision of the splice acceptor-βgeo-polyA sequence, the targeted allele is predicted to be a null allele of Gmnn. Excision by FLPe recombinase results in a targeted (floxed) allele that generates a wild-type mRNA. This floxed allele can be converted to a null allele in the presence of Cre, which excises exon 4. C57BL/6-Tg(Neurod2-Smo*A1)199Jols/J (Jax 008831) and B6.Cg-Tg(Nes-Cre)1Kln/J (Jax 003771) mice were obtained from Jackson laboratories.

### Brain isolation, sectioning and immunohisto-chemistry

Mice were euthanized by cardiac perfusion after anaesthesia according to institutionally approved protocols. Brains were processed overnight in 4% paraformaldehyde and embedded in paraffin, and matched 5 μm para-sagittal sections for each genotype were processed for immunohistochemistry (IHC) or stained with hematoxylin and eosin. IHC was as previously described [29] using the primary antibodies indicated, and slides were processed for Streptavidin-Peroxide detection using the Invitrogen Histostain-SP kit Cat No. #95-9943 LAB-SA detection system, following the manufacturer's protocol.

### Immunocytochemistry

Cells were fixed using 4% paraformaldehyde, washed with PBS containing 0.1% TritonX-100, blocked with PBS containing 1% donkey serum, 1% BSA and 0.1% TritonX-100, and incubated with the respective primary antibodies overnight at 4°C. The next day, the slides were washed as above, and secondary antibody staining was performed along with DAPI staining. The slides were mounted using Prolong Gold anti-fade (Life Technologies: P36931) and imaged using a confocal microscope, with all images taken at 20X magnification.

### Antibodies

Primary antibodies used for IHC, Immunocytochemistry, or Western blotting were Gmnn: sc-13015 (Santa Cruz), Ki67: MA1-90584 (ThermoFisher), Cleaved Caspase-3: #9661 (Cell Signaling), Cyclin D1: #134175 (Abcam), p27Kip1: #32034 (Abcam), Phospho-histone H3: 06-570 (Millipore), γH2AX : 05-636 (Millipore); total CHK2: #2662 (Cell Signaling Technology); Phospho-(T68) CHK2: BS4043 (Bioworld); Phospho-CHK1: #2341 (Cell Signaling Technology); Total CHK1: #2360 (Cell Signaling Technology); γTubulin: ab16504 (Abcam), GAPDH: G9545 (Sigma Aldrich).

### qRTPCR and immunoblotting

Total RNA was extracted with the Clontech Nucleospin RNA extraction kit (#740955.250), using the manufacturer's protocol. cDNA was synthesized using the Bio Rad iScript Reverse Transcriptase Supermix (#170-8841), followed by quantitative RT-PCR analysis using the Fast SYBR green mix from Applied Biosystems (#4385612), as previously described [34]. Primers used were: mouse Gmnn (F-GCAGTACATGGCGGAGGTAATC and R-GCTCCTGAGTCTTCCAGTTCTG), mouse α-Actin (F-CATTGCTGACAGGATGCAGAAGG and R-TGCTGGAAGGTGGACAGTGAGG), human Gmnn (F-CAGCCTTCTGCATCTGGATCTC and R-CCAGGGCTGGAAGTTGTAGATG), human GAPDH (F-GTCTCCTCTGACTTCAACAGCG and R-ACCACCCTGTTGCTGTAGCCAA), human α-Actin (F-CACCATTGGCAATGAGCGGTTC and R-AGGTCTTTGCGGATGTCCACGT). Protein lysate preparation and Western blotting was performed as previously described [30].

### Cell culture and siRNA transfection

The Daoy (ATCC HTB-186) cell line was grown in 10% FBS containing DMEM medium. Lipofectamine RNAi max reagent from Thermo Fisher Scientific was used for siRNA transfections following the manufacture's protocol. siRNA sequences are: siGem-2: AACUUCCAGCCCUGGGGUUAUUU [10], siGem-6: Target sequence GAAUAGUUCUGUCCCAAGA (siGENOME Human Gmnn Dharmacon), siGAPDH: UGGUUUACAUGUUCCAAUA (siGENOMEGapdh control Dharmacon).

### Growth curve analysis

Cells were counted and seeded at 600,000 cells/ml on Day 0 and transfected 24 hours later following the methods described in the earlier section. The cell numbers in each condition were counted and plotted on the growth curve.

### FACS analysis

1 million cells from each condition were pelleted, washed with PBS (without calcium and magnesium), re-suspended in FACS buffer (PBS plus 3% FBS, 0.04% Na-Azide and 1mM EDTA), and fixed by adding drops of ice cold 70% ethanol. The cells were stained with 1mg/ml propidium iodide (P4864; Sigma; 400μl) in 10 mls of PBS plus RNase A (500μl). Single cell suspensions of 1 million cells per condition were used for FACS analysis, with propidium iodide staining as a measure for DNA content and cell cycle analysis was performed using FloJo software.

### Cell cycle synchronization with Hydroxyurea

Daoy cells were counted and seeded at 600,000 cells/10 cm dish. 24 hours later cells were transfected with siRNAs targeting Gmnn (siGem-2 and siGem-6) or Gapdh as a control, using Lipofectamine RNAi max transfection protocol (Thermo Fisher). 25 nM of each siRNA was used. 12 hours later the cells were treated with 2 mM hydroxyurea (Sigma Aldrich H8627), to arrest the cells in the G1 phase of cell cycle, after [85]. After 14 hrs. of hydroxyurea treatment, cells were thoroughly washed with DMEM +10% FBS medium to remove any residual hydroxyurea, and were pelleted for propidium iodide staining and FACS analysis 48 hours after the HU release.

Etoposide treatment. 100nM final etoposide (E1383; Sigma Aldrich) dose was used in treating Daoy cells in comparison with a DMSO only treatment control. For ICC, positive control cells for γH2AX staining were generated by treating Daoy cells with 1 μM etoposide for 24 hours.

## SUPPLEMENTARY FIGURES AND TABLES


