# Supplementary material for: Geminin deficiency enhances survival in a murine medulloblastoma model by inducing apoptosis of preneoplastic granule neuron precursors
Source: Genes Cancer. 2017 Sep;8(9-10):725–44. doi: 10.18632/genesandcancer.157 (PMC5724806; doi:10.18632/genesandcancer.157)
Supplement: Supplementary file 1 [file ganc-08-725-s001.pdf]

Geminin deficiency enhances survival in a murine medulloblastoma model by inducing apoptosis of preneoplastic granule neuron precursors – Sankar etal

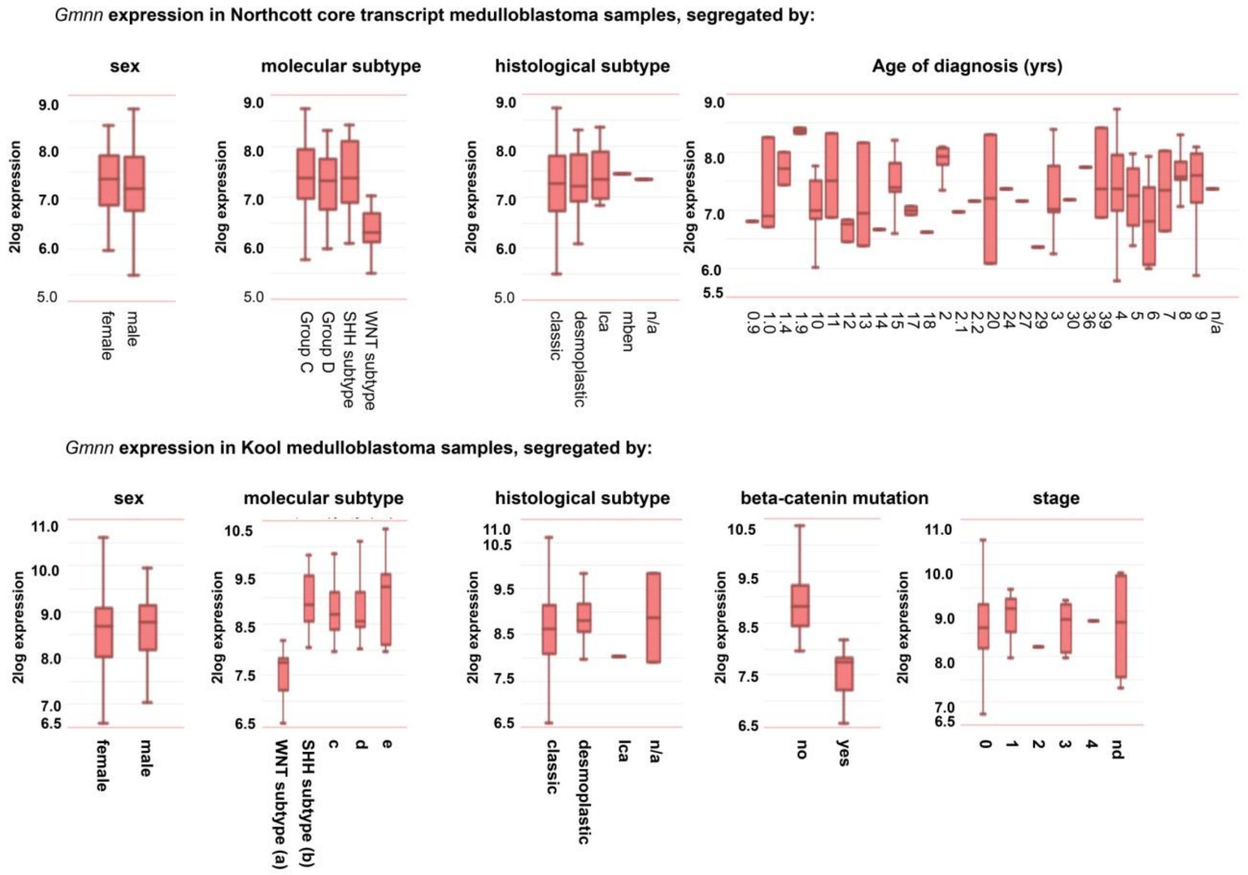

Figure S1: **Geminin expression levels in medulloblastomas do not correlate with tumor molecular or histological subtypes, staging, age of diagnosis, or sex.** Geminin expression levels were analyzed in two human medulloblastoma datasets by using the R2 Megasampler tool (see Methods; datasets used were Northcott core transcript, n=103, GSE21140 and Kool, n=62, GSE10327) and did not segregate based upon sex, molecular or histological subtype, age of diagnosis, or stage. *Gmnn* is on human chr6 and expression levels are lower in the Wnt and β-catenin mutated subtypes, congruent with monosomy 6 in the Wnt tumor subclass.

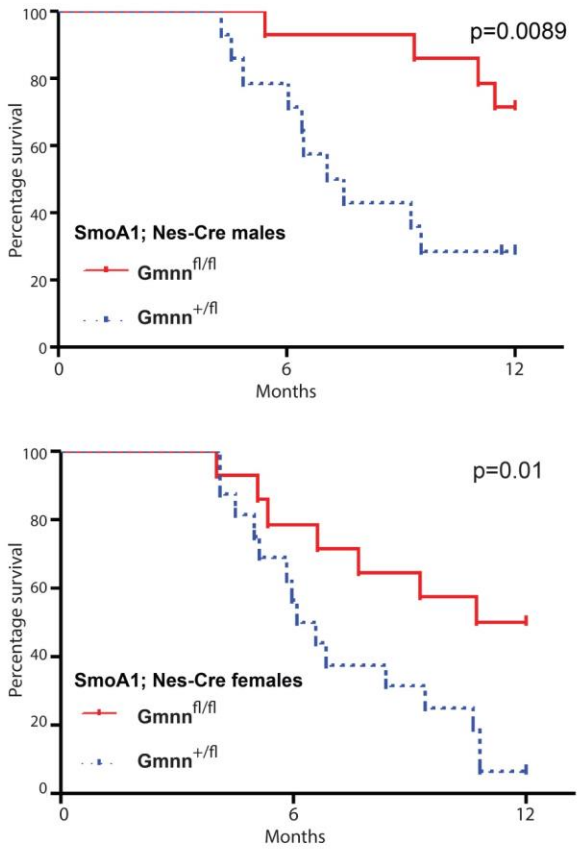

Figure S2: Geminin loss of function enhances survival in the SmoA1 medulloblastoma model. Kaplan-Meier survival plot is shown for cohorts of male or female mice hemizygous for both the Nes-Cre and SmoA1 transgenes and either heterozygous or homozygous for the floxed Gem allele. Animals were euthanized by transcardial perfusion when they exhibited neurologic impairment or morbidity or at the end of the study and presence or absence of tumors assessed by sectioning and histology.

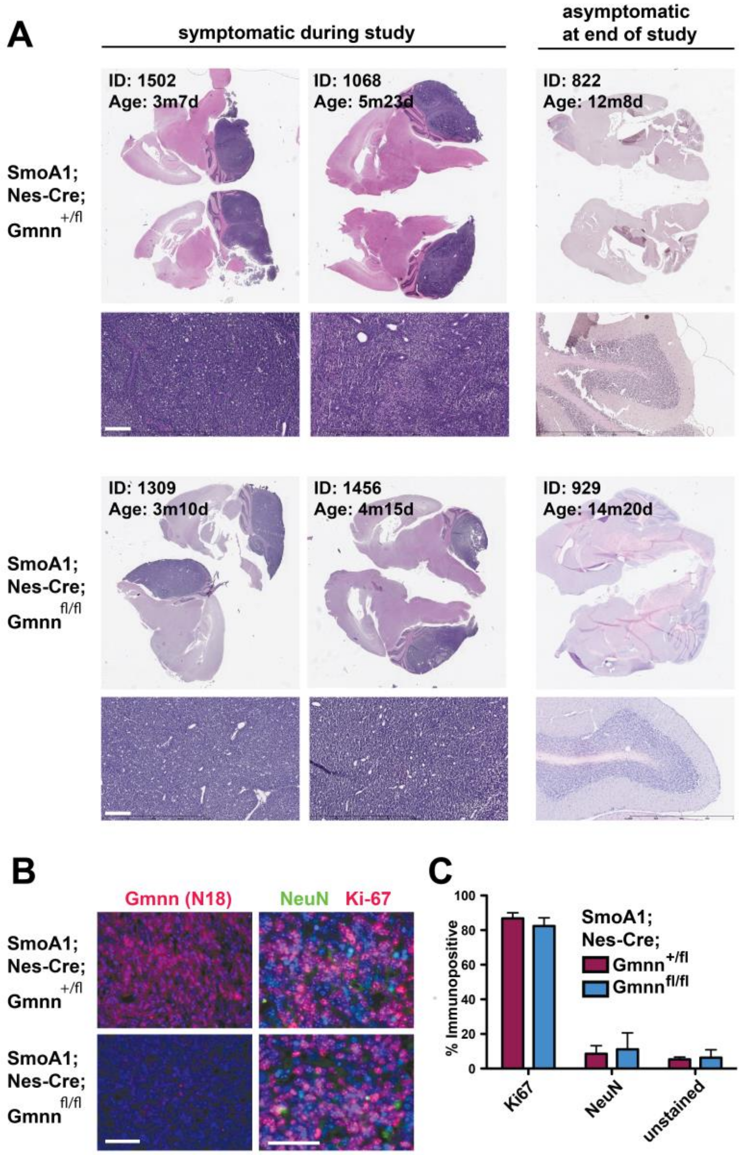

Figure S3: Histology and molecular marker expression in SmoA1 tumors. (**A**) Cohorts of mice with the SmoA1; Nes-Cre; $Gmnn^{+/fl}$ and SmoA1; Nes-Cre; $Gmnn^{fl/fl}$ genotypes were generated and tracked for one year. Mice that developed clinical symptoms of medulloblastoma (enlarged posterior fossa, tilted head, hunched posture, ataxia) and animals that remained asymptomatic through the end of the study were euthanized and histological sectioning of the brain was performed. Examples are shown, with identification number and age at the time euthanized indicated. Parasaggital sections of all symptomatic animals revealed large, bilateral tumor masses, while very few animals that remained asymptomatic

through the end of the study exhibited a visible tumor mass. Lower panels: higher magnification views through the tumor or cerebellum. Tumor size and histology appeared similar between the two genotypes. Scale bar=200µm. (**B**) For each genotype, three tumors were also analyzed by frozen sectioning and immunofluorescence for Gmnn, Ki67, and NeuN, with DAPI (blue) counterstaining of all nuclei. Scale bars=50µm, with (**C**) numbers of Ki67 or NeuN immunopositive cells scored for three sections through each tumor and expressed as a percentage +/- stdev. Gmnn protein levels were strongly diminished in the SmoA1; Nes-Cre; $Gmnn^{fl/fl}$ tumors, while similar fractions of cells expressing Ki67 or NeuN were seen for both genotypes.

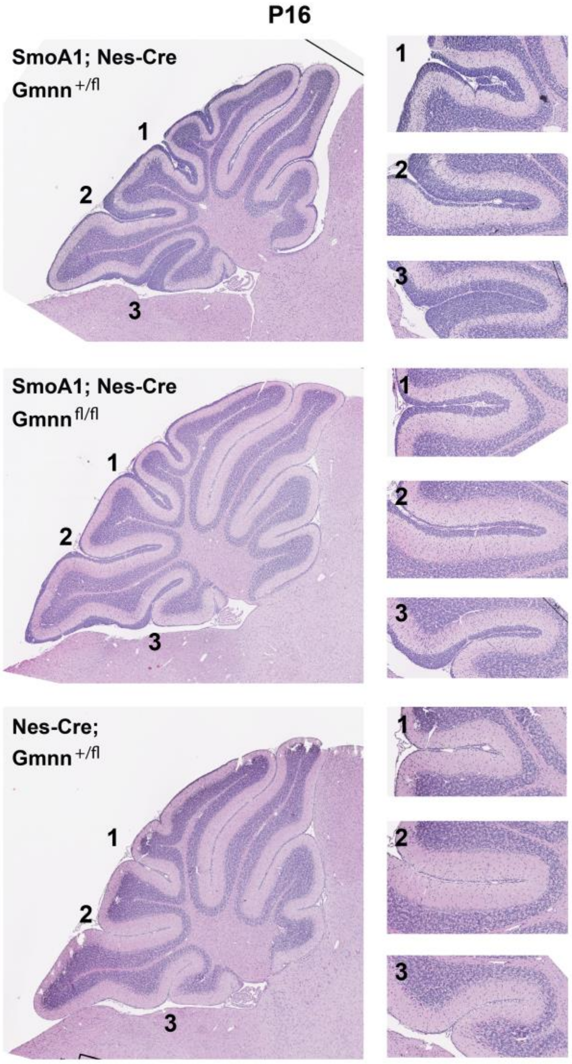

Figure S4: Geminin loss reduces numbers of persisting preneoplastic GNPs in SmoA1 mice.

Animals hemizygous for the SmoA1 and Nes-Cre transgenes and heterozygous for the Gmnn floxed allele (SmoA1; Nes-Cre; $Gmnn^{+/fl}$) retained much thicker EGL regions in P16 cerebellar sections than those seen in SmoA1; Nes-Cre; $Gmnn^{fl/fl}$ homozygous animals, with animals lacking the SmoA1 transgene shown as an additional control.

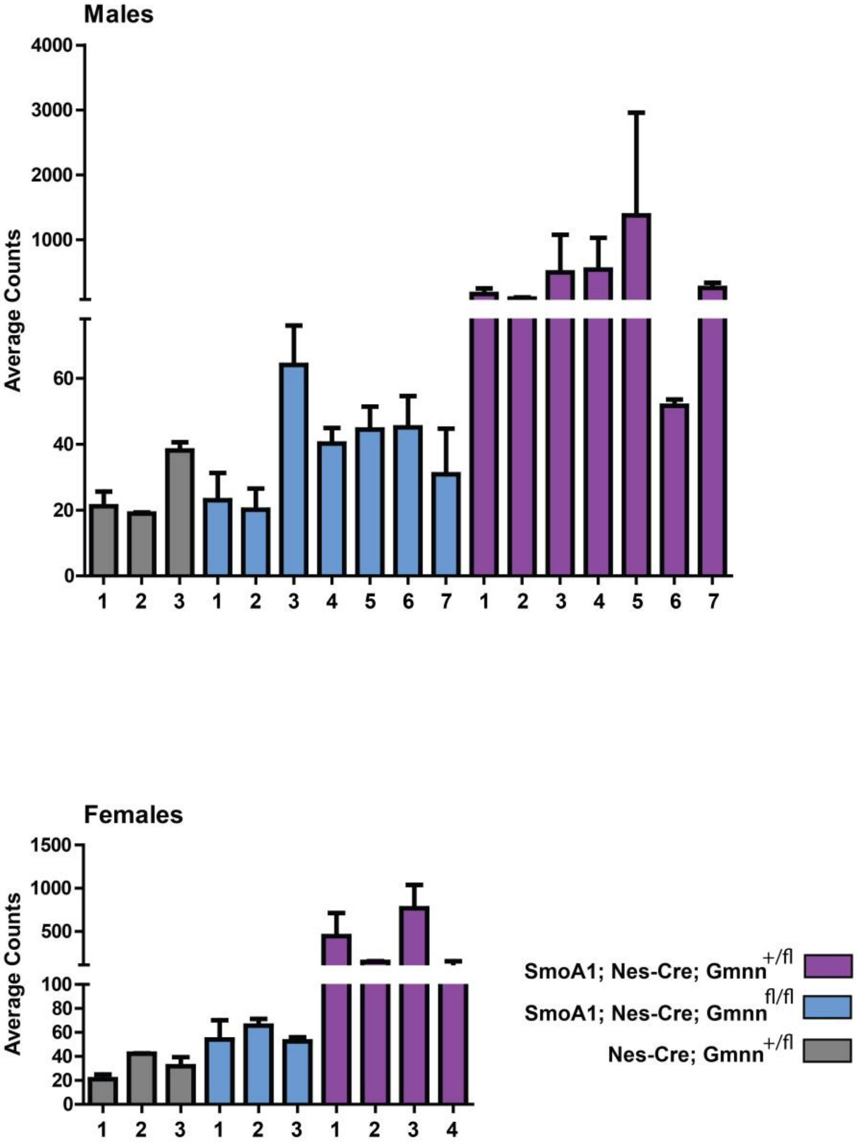

Figure S5: Quantitation of GNPs at the cerebellar surface in individual P28 animals, comparing animals hemizygous for the SmoA1 and Nes-Cre transgenes and heterozygous versus homozygous for the Gmnn floxed allele, with animals lacking the SmoA1 transgene as an additional control. Quantitative data (summarized for all animals in **Fig. 3D**) was obtained as averaged counts/section from 4 matched serial sections per brain, and is shown as averaged counts for each animal assayed. SmoA1; Nes-Cre; $Gmnn^{fl/fl}$ homozygous P28 animals had six-fold fewer pGNPs than SmoA1; Nes-Cre; $Gmnn^{+/fl}$ heterozygotes.

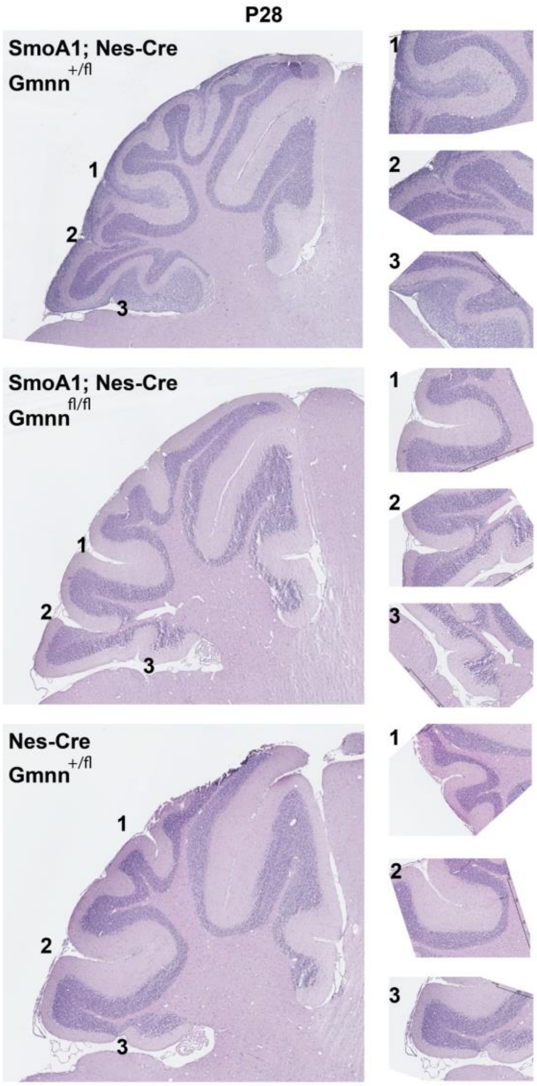

Figure S6: Example of severe dysplasia seen in some P28 animals hemizygous for the Nes-Cre and SmoA1 transgenes and heterozygous for the floxed Gmnn allele. This was not observed in any of the Nes-Cre; SmoA1 animals homozygous for the floxed Gmnn allele. Example of one representative animal for each genotype is shown.

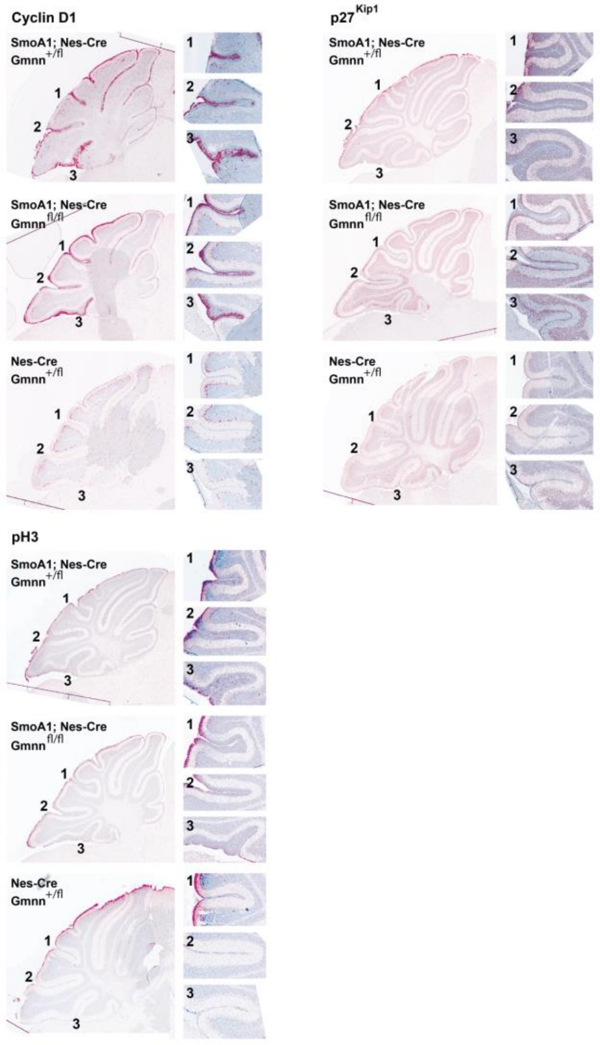

Figure S7: Immunohistochemistry for Cyclin D1, p27^{Kip1}, and pH3 on P14 cerebellar sections from animals hemizygous for the Nes-Cre and SmoA1 transgenes and either heterozygous or homozygous for the Gmnn floxed allele. No significant differences in the relative frequency of GNPs immunopositive for these antibodies was observed between animals heterozygous versus homozygous for the Gmnn floxed allele.

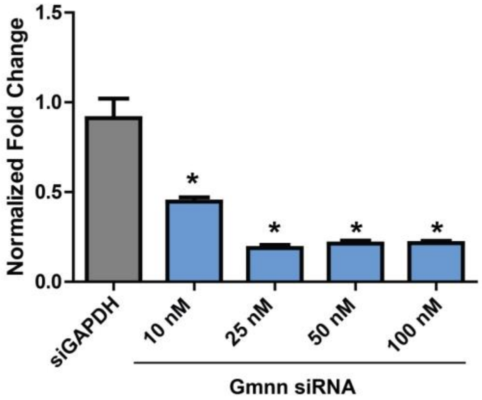

Figure S8: Gmnn siRNA dose response in Daoy cells. Daoy cells were transfected with increasing concentrations of a Gmnn siRNA (#2) as shown. After 72 hrs., Gmnn mRNA levels were analyzed by qRTPCR, represented as normalized fold change relative to the control (siGAPDH). Subsequent experiments used the lowest dose of Gmnn siRNA #2 that provided effective knockdown (25 nM). *<0.05 was defined by two-tailed student's t-test.

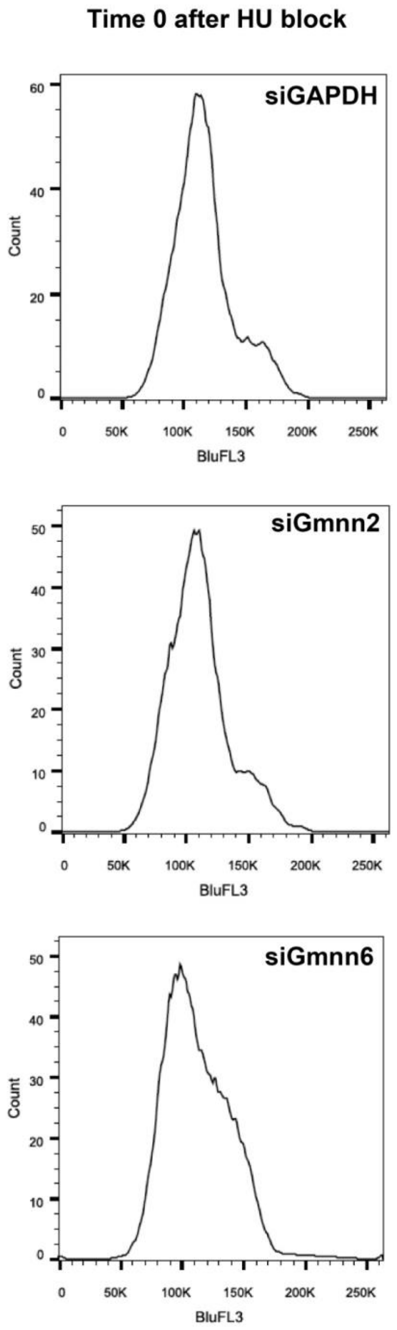

Figure S9: Cell cycle synchronization of Daoy cells. Synchronization with 100 nM hydroxyurea (HU) was performed for 14 hrs as described (see Methods). Time 0 after release from HU was analyzed by flow cytometry analysis with PI stained cells. In all three knockdown conditions, cells are predominantly synchronized in G1/S of cell cycle.

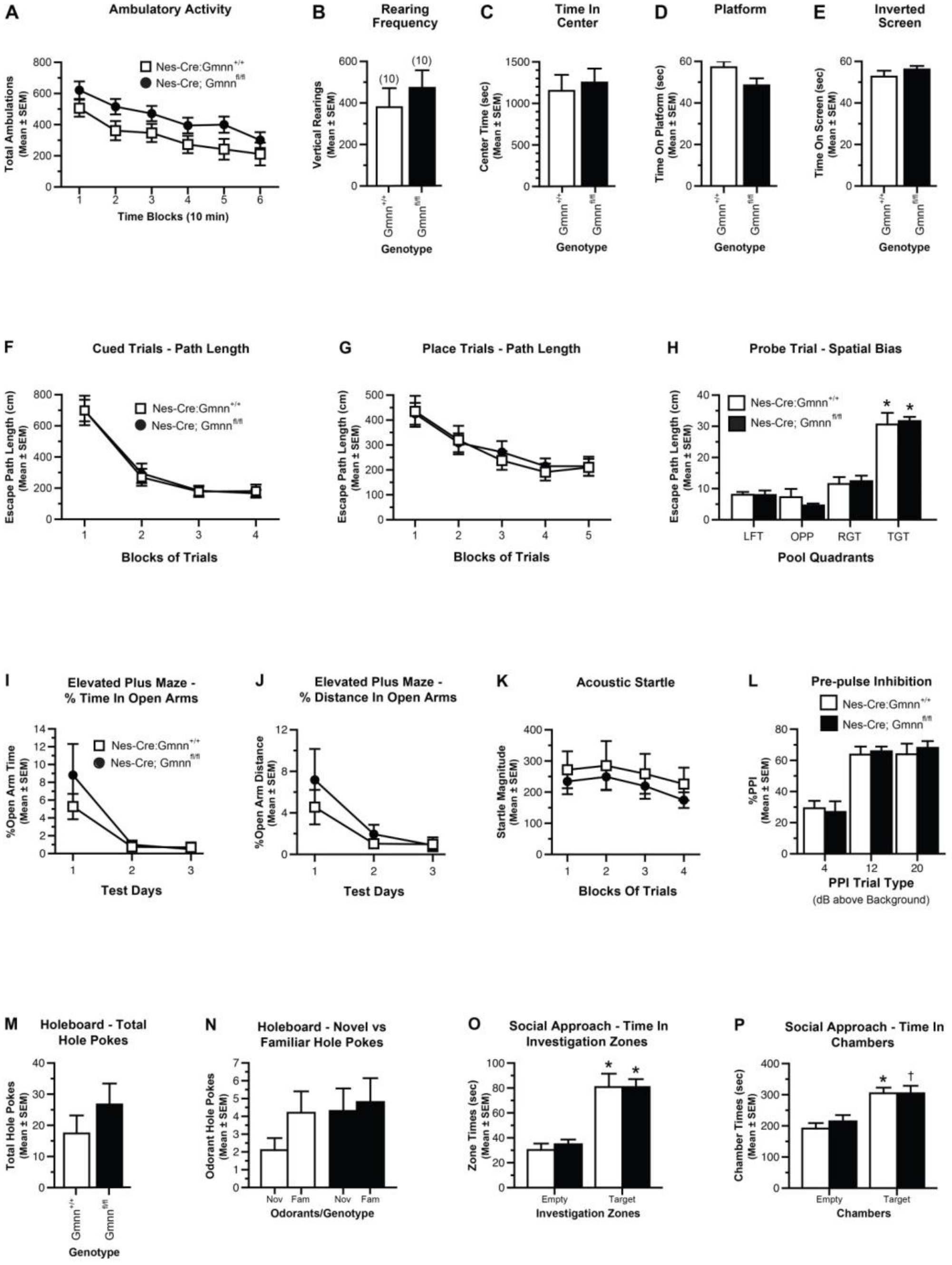

Figure S10: Behavioral testing failed to reveal a definitive phenotype in Nes-Cre; Gmnn conditional loss of function mice. To characterize any behavioral phenotypes resulting from Nes-Cre-mediated Geminin conditional loss of function, we generated cohorts of 3-4 month old mice that were hemizygous for the Nes-Cre transgene and either wild-type for Gmnn (Nes-Cre; $Gmnn^{+/+}$; n=10;

males=5/females=5) or homozygous for the floxed Gmnn allele (Nes-Cre; $Gmnn^{fl/fl}$)(n=10; males=5/females=5). These were evaluated utilizing a range of behavioral analyses, as described in the Supplemental Methods and Results. **(A-C)** Results from a 1-h locomotor activity test showed that the $Gmnn^{+/+}$ and $Gmnn^{fl/fl}$ groups did not differ in terms of ambulatory activity **(A)**, vertical rearing frequency **(B)** or in time spent in the center of the test field **(C)**. **(D-E)** The $Gmnn^{+/+}$ and $Gmnn^{fl/fl}$ mice also did not differ significantly on the platform **(D)** or inverted screen **(E)** tests, which were part of the battery of sensorimotor measures. **(F-H)** Testing in the Morris water maze did not reveal any cognitive deficits in the $Gmnn^{+/+}$ and $Gmnn^{fl/fl}$ mice in terms of acquisition performance during the cued **(F)** or place (spatial) learning **(G)** trials. Likewise, the groups both showed intact retention during the probe trial by exhibiting a spatial bias for the target quadrant that had contained the submerged platform **(H)**. This was documented by each group spending significantly more time in the target quadrant versus the times spent in each of the other quadrants (*$p<0.008$). (**I-J**) Elevated plus maze test assessed time spent and distance travelled in the open arms relative to total time/distance in all of the arms and did not detect significant differences. **(K-L)** Analysis of the acoustic startle/PPI data indicated that the groups did not differ in terms of the magnitude of the ASR **(K)**, nor in %PPI levels **(L)**, suggesting that sensorimotor reactivity and gating did not differ in the $Gmnn^{+/+}$ and $Gmnn^{fl/fl}$ mice. **(M-N)** Analysis of the data from the holeboard exploration/olfactory preference test showed that the $Gmnn^{+/+}$ and $Gmnn^{fl/fl}$ groups did not differ in terms of total exploratory hole pokes **(M)** or in terms of poke frequencies for corner holes that contained a novel versus a familiar odorant **(N)**. **(O-P)** The results from the social approach test did not provide evidence of abnormal behaviors on the part of the $Gmnn^{fl/fl}$ mice. Specifically, the $Gmnn^{+/+}$ and $Gmnn^{fl/fl}$ groups showed similar degrees of sociability by exhibiting significantly increased investigation zone times surrounding the stimulus mouse (**O**) relative to the times in the zones around the empty withholding cage (*$p<0.00005$), and each group spent more time in the chamber containing the stimulus mouse (**P**) compared to the empty chamber (*$p=0.003$; †$p=0.035$).

Tables S1-S3: Genes whose expression is most strongly correlated with that of Geminin in 103 medulloblastoma samples were defined for the Northcott core transcript dataset (GSE21140) using the R2 Megasampler (http://hgserver1.amc.nl/cgi-bin/r2/main.cgi). GO terms for genes with Gmnn co-correlated (**Table S1**) and anti-correlated (**Table S2**) expression and genes with Gmnn co-correlated expression (**Table S3**) are shown.

Geminin expression co-correlated GO terms

Enrichment by Process Networks

#	Networks	p-value	FDR
1	Cell cycle_Core	1.37E-15	5.60E-14
2	Cell cycle_Mitosis	1.08E-12	2.21E-11
3	Cytoskeleton_Spindle microtubules	1.07E-11	1.47E-10
4	Cell cycle_S phase	2.65E-11	2.72E-10
5	Cell cycle_G2-M	1.81E-08	1.48E-07
6	DNA damage_MMR repair	6.26E-05	4.28E-04
7	Transcription_mRNA processing	1.00E-03	5.86E-03
8	DNA damage_DBS repair	1.47E-03	7.52E-03
9	Cell cycle_Meiosis	7.19E-03	3.28E-02
10	Transcription_Chromatin modification	1.37E-02	5.15E-02

Enrichment by GO Molecular Functions

#	Molecular functions	p-value	FDR
1	nucleotide binding	2.47E-07	1.57E-05
2	protein binding	3.31E-07	1.57E-05
3	ATP binding	3.32E-07	1.57E-05
4	protein serine/threonine kinase activity	6.09E-07	2.16E-05
5	damaged DNA binding	2.01E-06	5.70E-05
6	transferase activity, transferring phosphorus-containing groups	4.94E-05	1.17E-03
7	DNA binding	7.62E-05	1.54E-03
8	kinetochore binding	8.73E-05	1.55E-03
9	kinase activity	1.05E-04	1.66E-03
10	single-stranded DNA binding	2.32E-04	3.30E-03

Enrichment by GO Processes

#	Processes	p-value	FDR
1	cell cycle	1.20E-29	5.48E-27
2	mitotic cell cycle	9.85E-25	2.26E-22
3	cell division	3.02E-18	4.61E-16
4	mitosis	1.29E-17	1.48E-15
5	chromosome segregation	6.43E-11	5.89E-09
6	nucleosome assembly	1.59E-09	1.22E-07
7	response to DNA damage stimulus	4.78E-09	3.13E-07
8	DNA repair	1.19E-08	6.80E-07
9	G1/S transition of mitotic cell cycle	2.03E-08	1.03E-06
10	attachment of spindle microtubules to kinetochore	2.66E-08	1.22E-06

Enrichment by Pathway Maps

#	Maps	p-value	FDR
1	Cell cycle_Role of APC in cell cycle regulation	2.59E-11	1.14E-09
2	Cell cycle_The metaphase checkpoint	6.36E-11	1.40E-09
3	Cell cycle_Start of DNA replication in early S phase	1.38E-07	2.02E-06
4	Cell cycle_Chromosome condensation in prometaphase	1.20E-06	1.32E-05
5	Cell cycle_Role of Nek in cell cycle regulation	6.98E-06	5.81E-05
6	Cell cycle_Spindle assembly and chromosome separation	7.92E-06	5.81E-05
7	DNA damage_Mismatch repair	6.29E-05	3.95E-04
8	Reproduction_Progesterone-mediated oocyte maturation	5.16E-04	2.84E-03
9	Cell cycle_Initiation of mitosis	4.37E-03	2.08E-02
10	DNA damage_ATM / ATR regulation of G2 / M checkpoint	4.72E-03	2.08E-02

Enrichment by GO Localizations

#	Localizations	p-value	FDR
1	nucleus	9.71E-22	1.02E-19
2	condensed chromosome kinetochore	2.27E-16	1.19E-14
3	nucleoplasm	4.78E-15	1.67E-13
4	chromosome, centromeric region	6.69E-14	1.69E-12
5	chromosome	8.06E-14	1.69E-12
6	kinetochore	1.65E-13	2.89E-12
7	cytosol	5.19E-08	7.78E-07
8	spindle	1.72E-07	2.26E-06
9	condensed chromosome, centromeric region	4.72E-07	5.50E-06
10	centrosome	1.36E-06	1.43E-05

Geminin expression anti-correlated GO terms

#	Networks	p-value	FDR
1	Transport_Calcium transport	2.363E-02	6.633E-01
2	Neurophysiological process_Transmission of nerve impulse	3.247E-02	6.633E-01
3	Transport_Sodium transport	4.133E-02	6.633E-01
4	Reproduction_Male sex differentiation	5.145E-02	6.633E-01
5	Signal transduction_Neuropeptide signaling pathways	5.871E-02	6.633E-01
6	Muscle contraction	7.628E-02	6.633E-01
7	Inflammation_Innate inflammatory response	8.476E-02	6.633E-01
8	Cell adhesion_Synaptic contact	8.804E-02	6.633E-01
9	Neurophysiological process_Olfactory transduction	1.038E-01	6.633E-01
10	Reproduction_Gonadotropin regulation	1.052E-01	6.633E-01
Enrichment by GO Molecular Functions			
#	Molecular functions	p-value	FDR
1	enzyme activator activity	2.048E-10	3.626E-08
2	lipid binding	2.676E-05	2.368E-03
3	ion channel activity	2.752E-04	1.623E-02
4	voltage-gated calcium channel activity	4.805E-04	2.111E-02
5	high voltage-gated calcium channel activity	6.702E-04	2.111E-02
6	calcium channel activity	7.156E-04	2.111E-02
7	protein-hormone receptor activity	8.353E-04	2.112E-02
8	5-hydroxy-6E,8Z,11Z,14Z-icosatetraenoic acid binding	4.385E-03	4.085E-02
9	somatostatin receptor binding	4.385E-03	4.085E-02
10	fucokinase activity	4.385E-03	4.085E-02
Enrichment by GO Processes			
#	Processes	p-value	FDR
1	positive regulation of peptide secretion	2.740E-07	1.982E-04
2	negative regulation of heart rate	3.621E-07	1.982E-04
3	cytoskeletal anchoring at plasma membrane	8.041E-07	2.201E-04
4	negative regulation of hormone secretion	8.041E-07	2.201E-04
5	smooth muscle contraction involved in micturition	2.376E-06	5.203E-04
6	regulation of vasodilation	3.251E-06	5.490E-04
7	adult walking behavior	3.510E-06	5.490E-04
8	transmission of nerve impulse	5.213E-06	6.601E-04
9	negative regulation of renal sodium excretion	5.668E-06	6.601E-04
10	calcium ion import	6.028E-06	6.601E-04
Enrichment by Pathway Maps			
#	Maps	p-value	FDR
1	Neurophysiological process_ACM regulation of nerve impulse	3.104E-04	2.917E-02
2	Neurophysiological process_Kappa-type opioid receptor in transmission of ner	3.409E-03	1.242E-01
3	Nicotine signaling in glutamatergic neurons	4.116E-03	1.242E-01
4	Keratan sulfate metabolism p.2	5.715E-03	1.242E-01
5	Apoptosis and survival_Apoptotic TNF-family pathways	6.604E-03	1.242E-01
6	Ascorbate metabolism	5.149E-02	1.867E-01
7	Cytoskeleton remodeling_Alpha-1A adrenergic receptor-dependent inhibition c	5.428E-02	1.867E-01
8	Neurophysiological process_Olfactory transduction	5.706E-02	1.867E-01
9	Role of ZNF202 in regulation of expression of genes involved in atherosclerosi	5.982E-02	1.867E-01
10	Taurine and hypotaurine metabolism	6.259E-02	1.867E-01
Enrichment by GO Localizations			
#	Localizations	p-value	FDR
1	plasma membrane	7.270E-08	9.160E-06
2	integral to membrane	3.243E-06	2.043E-04
3	membrane	2.516E-05	1.057E-03
4	collagen	6.476E-05	2.040E-03
5	integral to plasma membrane	1.394E-04	3.514E-03
6	voltage-gated calcium channel complex	2.292E-04	4.813E-03
7	dendrite terminus	5.940E-04	1.069E-02
8	proteinaceous extracellular matrix	2.719E-03	4.283E-02
9	postsynaptic density	3.718E-03	4.329E-02
10	collagen type XIII	4.123E-03	4.329E-02

Geminin expression co-correlated genes in medulloblastoma

probeset	HUGO	R-value	R-pvalue
2898597	GMNN	1	0
2914777	TTK	0.829	1.38E-23
2980241	FBXO5	0.829	1.87E-23
2894663	PAK1IP1	0.79	1.22E-19
2451200	UBE2T	0.779	8.74E-19
2813442	CENPH	0.77	4.40E-18
3776139	NDC80	0.761	2.20E-17
2793951	HMGB2	0.758	3.08E-17
2411228	STIL	0.755	4.56E-17
2494484	NCAPH	0.755	4.87E-17
2416218	ITGB3BP	0.752	6.37E-17
2973168	ECHDC1	0.751	7.08E-17
2929036	LTV1	0.75	8.40E-17
2780172	CENPE	0.747	1.37E-16
2364438	NUF2	0.741	3.27E-16
2388219	EXO1	0.734	1.03E-15
2748163	MND1	0.731	1.44E-15
2783715	MAD2L1	0.73	1.52E-15
2813414	CCNB1	0.729	1.84E-15
2921374	RPF2	0.728	1.91E-15
2577896	MCM6	0.726	2.65E-15
2468920	CPSF3	0.722	5.01E-15
2742985	PLK4	0.721	5.17E-15
2652675	ECT2	0.72	5.86E-15
2951300	TAF11	0.72	5.49E-15
3728964	PRR11	0.719	5.85E-15
2908572	CDC5L	0.717	8.61E-15
2425447	DPH5	0.716	8.77E-15
3595979	CCNB2	0.715	1.06E-14
2604254	HJURP	0.713	1.35E-14
2522212	SGOL2	0.711	1.72E-14
2570616	BUB1	0.711	1.78E-14
3011454	DBF4	0.709	2.03E-14
2665572	SGOL1	0.707	2.68E-14
2752725	NEIL3	0.704	3.97E-14
2966232	COQ3	0.703	4.75E-14
2919873	QRSL1	0.7	7.25E-14
3658925	ORC6	0.7	6.74E-14
2908100	POLH	0.699	7.79E-14
2893721	RIOK1	0.696	1.19E-13
2417528	DEPDC1	0.695	1.28E-13
2951674	SRPK1	0.695	1.27E-13
3331903	FAM111B	0.694	1.34E-13
2481142	MSH6	0.693	1.54E-13
2958670	RAB23	0.691	1.90E-13
2450345	KIF14	0.69	2.26E-13
2458742	LIN9	0.689	2.32E-13
3603408	PSMA4	0.688	2.62E-13
2346399	CDC7	0.686	3.70E-13
2412799	ORC1	0.685	4.11E-13
2334098	KIF2C	0.684	4.34E-13
2620256	KIF15	0.684	4.58E-13
3354799	CHEK1	0.683	4.95E-13
2553730	MTIF2	0.68	6.97E-13
2859667	CENPK	0.68	6.92E-13
2908052	POLR1C	0.68	6.92E-13
3044753	LSM5	0.68	7.04E-13
2548970	SRSF7	0.679	7.38E-13
2905118	SRSF3	0.679	7.27E-13
2905469	RNF8	0.679	7.08E-13
2971139	ZUFSP	0.677	9.08E-13
3910785	AURKA	0.677	8.72E-13
2916952	CASP8AP2	0.676	1.05E-12
2519140	ZC3H15	0.675	1.08E-12
2965739	MMS22L	0.675	1.17E-12
2923819	HSF2	0.674	1.29E-12
2347023	CCDC18	0.672	1.57E-12
2893847	SNRNP48	0.671	1.82E-12
3484641	BRCA2	0.671	1.65E-12
2926476	TBPL1	0.67	1.79E-12
2931683	C6orf211	0.67	1.81E-12
2978957	KATNA1	0.67	1.81E-12
2779486	H2AFZ	0.669	2.03E-12
3168508	MELK	0.668	2.24E-12
3428845	PARPBP	0.668	2.27E-12
2840002	SPDL1	0.667	2.52E-12
2975655	MTFR2	0.667	2.44E-12
2826064	SRFBP1	0.666	2.67E-12
2970532	HDAC2	0.665	3.17E-12
2515369	HAT1	0.664	3.33E-12
3599811	KIF23	0.662	4.47E-12
3590086	RAD51	0.661	4.94E-12
2963407	SYNCRIP	0.66	5.20E-12
2454444	NEK2	0.659	5.86E-12
2966371	CCNC	0.659	5.83E-12
2516023	CDCA7	0.658	6.55E-12
2801526	CCT5	0.658	6.60E-12
2334404	NASP	0.657	7.07E-12
2330899	UTP11L	0.655	9.62E-12
2952679	GLO1	0.655	9.68E-12
2518743	NUP35	0.654	9.86E-12
3248289	CDK1	0.654	9.94E-12
2947073	HIST1H1B	0.652	1.26E-11
2997376	ANLN	0.649	1.69E-11
2488078	MPHOSPH1	0.648	1.97E-11
3765580	BRIP1	0.648	1.90E-11
2732611	MRPL1	0.647	2.22E-11
2967550	ATG5	0.647	2.15E-11
2423597	DNTTIP2	0.646	2.27E-11
3515965	DIAPH3	0.646	2.35E-11
3590014	CASC5	0.646	2.30E-11
2963707	RARS2	0.645	2.47E-11
3607510	FANCI	0.645	2.46E-11
2773997	NUP54	0.643	3.00E-11
3401804	RAD51AP1	0.643	3.05E-11
3563395	POLE2	0.642	3.56E-11
2378937	DTL	0.641	4.02E-11
2812120	CWC27	0.639	4.68E-11

probeset	HUGO	R-value	R-pvalue
2427074	PSMA5	0.638	5.01E-11
3080283	XRCC2	0.638	5.01E-11
2858668	ERCC8	0.637	5.70E-11
2679796	THOC7	0.636	6.36E-11
2805786	TARS	0.636	6.08E-11
2811745	IPO11	0.636	6.04E-11
3003143	MRPS17	0.636	6.16E-11
2746269	LSM6	0.635	6.52E-11
3291435	RTKN2	0.635	6.60E-11
2380554	RRP15	0.634	7.33E-11
2740005	LARP7	0.634	7.61E-11
2491935	PTCD3	0.632	9.30E-11
3881443	TPX2	0.632	9.09E-11
2553970	PNPT1	0.631	9.90E-11
2934089	WTAP	0.63	1.13E-10
3342426	C11orf82	0.629	1.22E-10
4012142	ERCC6L	0.627	1.57E-10
2818454	XRCC4	0.626	1.58E-10
3936913	CDC45	0.626	1.62E-10
2730531	UTP3	0.625	1.83E-10
2815455	UTP15	0.623	2.12E-10
2353773	TTF2	0.622	2.31E-10
2443518	METTL18	0.622	2.45E-10
2858592	DEPDC1B	0.622	2.32E-10
2948564	MDC1	0.622	2.33E-10
2957126	MCM3	0.622	2.34E-10
2954678	XPO5	0.621	2.58E-10
3005069	ZNF92	0.621	2.68E-10
3539147	SNAPC1	0.62	2.97E-10
2746024	ABCE1	0.619	3.03E-10
2971378	GOPC	0.619	3.03E-10
2980812	TFB1M	0.619	3.04E-10
2916390	ORC3	0.618	3.21E-10
2954005	MRPS10	0.618	3.26E-10
3019401	ZNF277	0.618	3.23E-10
3200611	HAUS6	0.618	3.24E-10
3433747	RFC5	0.618	3.21E-10
2593352	GTF3C3	0.617	3.65E-10
2923359	ASF1A	0.617	3.73E-10
3257338	KIF20B	0.615	4.44E-10
2838201	PTTG1	0.614	4.84E-10
3367338	KIF18A	0.614	4.84E-10
3590388	NUSAP1	0.612	6.06E-10
3775842	TYMS	0.611	6.26E-10
2639874	UMPS	0.61	6.78E-10
2847710	FASTKD3	0.61	6.74E-10
2714955	TACC3	0.609	7.52E-10
3793588	TIMM21	0.609	7.58E-10
2704188	PDCD10	0.608	7.97E-10
3468261	NUP37	0.608	7.96E-10
2788143	ANAPC10	0.607	9.13E-10
3367036	CCDC34	0.606	9.88E-10
3619945	OIP5	0.606	9.47E-10
2417737	LRRC40	0.603	1.34E-09
3258444	CEP55	0.603	1.29E-09
2331974	ZNF684	0.601	1.52E-09
2662020	RAD18	0.601	1.52E-09
2863049	POC5	0.601	1.56E-09
2950668	DAXX	0.601	1.56E-09
2986546	PDCD2	0.601	1.51E-09
3523855	TEX30	0.601	1.52E-09
2937984	TBP	0.6	1.69E-09
3013952	ACN9	0.6	1.60E-09
3854218	HAUS8	0.6	1.60E-09
2393816	C1orf174	0.599	1.77E-09
2566740	TXNDC9	0.598	1.93E-09
2673085	CDC25A	0.598	1.99E-09
3485074	RFC3	0.598	1.94E-09
2521278	CCDC150	0.597	2.14E-09
2636272	GTPBP8	0.597	2.13E-09
2939814	RPP40	0.597	2.11E-09
2969289	WASF1	0.597	2.17E-09
3784670	C18orf21	0.597	2.07E-09
3991109	MST4	0.597	2.17E-09
2727976	CEP135	0.596	2.36E-09
2945645	TDP2	0.596	2.34E-09
2489806	MRPL19	0.595	2.46E-09
2902178	TCF19	0.595	2.50E-09
3045047	RP9	0.595	2.55E-09
3653072	PLK1	0.595	2.43E-09
2901913	TUBB	0.594	2.64E-09
2643157	CDV3	0.593	2.86E-09
2900059	HIST1H2BM	0.593	2.98E-09
2904248	SNRPC	0.593	2.88E-09
3047963	PSMA2	0.593	2.89E-09
2497892	MRPS9	0.592	3.04E-09
2559696	TPRKB	0.592	3.13E-09
2564599	MRPS5	0.592	3.21E-09
2733928	COPS4	0.592	3.22E-09
3065963	ORC5	0.592	3.14E-09
3400190	CCDC77	0.592	3.23E-09
2924619	TRMT11	0.591	3.38E-09
3608298	BLM	0.591	3.31E-09
2424148	ALG14	0.59	3.55E-09
2693149	SNX4	0.59	3.55E-09
2733767	ENOPH1	0.59	3.56E-09
2855614	C5orf34	0.59	3.60E-09
2897576	E2F3	0.59	3.56E-09
3044129	GGCT	0.59	3.54E-09
3984655	CENPI	0.59	3.64E-09
2768145	COMMD8	0.588	4.19E-09
2815965	HMGCR	0.588	4.22E-09
2868283	RIOK2	0.588	4.21E-09
3056414	RFC2	0.588	4.05E-09
3536336	CDKN3	0.588	4.12E-09
3639031	PRC1	0.588	4.06E-09
3505937	CENPJ	0.587	4.35E-09
2555630	CCT4	0.586	4.78E-09

probeset	HUGO	R-value	R-pvalue
3042421	HNRNPA2B1	0.586	4.95E-09
3236395	HSPA14	0.586	4.85E-09
2396480	EXOSC10	0.585	5.53E-09
2616018	CNOT10	0.584	5.83E-09
3129149	PBK	0.584	5.62E-09
3290210	ZWINT	0.584	5.78E-09
2940987	SLC35B3	0.583	6.12E-09
3458133	PRIM1	0.581	7.21E-09
2469252	RRM2	0.58	8.18E-09
2689208	NAA50	0.58	7.77E-09
2709062	TRA2B	0.58	7.79E-09
2745220	ZNF330	0.579	8.80E-09
2860666	TAF9	0.579	8.57E-09
3269662	BCCIP	0.579	8.60E-09
2401275	HNRNPR	0.578	9.80E-09
3591704	WDR76	0.577	1.02E-08
2333136	CDC20	0.576	1.16E-08
3082181	NCAPG2	0.576	1.09E-08
2363902	DUSP12	0.575	1.20E-08
2523144	NOP58	0.575	1.24E-08
2610241	FANCD2	0.575	1.19E-08
3235789	MCM10	0.575	1.20E-08
3779684	PSMG2	0.575	1.19E-08
2547716	FAM98A	0.574	1.29E-08
2548871	HNRPLL	0.574	1.33E-08
2862716	GFM2	0.574	1.32E-08
2915491	CYB5R4	0.574	1.26E-08
2841528	BNIP1	0.573	1.38E-08
2902559	CSNK2B	0.573	1.38E-08
3090697	CDCA2	0.573	1.35E-08
3788049	SKA1	0.573	1.40E-08
2728224	SRP72	0.572	1.50E-08
2738723	HADH	0.572	1.46E-08
2823820	WDR36	0.572	1.55E-08
2339414	USP1	0.571	1.64E-08
2412988	SELRC1	0.571	1.63E-08
3097152	MCM4	0.571	1.55E-08
2917767	MANEA	0.57	1.77E-08
2574884	IWS1	0.569	1.94E-08
2653902	ZNF639	0.569	1.86E-08
3004628	ZNF107	0.569	1.95E-08
3716893	ATAD5	0.569	1.83E-08
2402068	SYF2	0.568	1.97E-08
2522439	BZW1	0.568	2.08E-08
2853768	NUP155	0.568	2.03E-08
3073597	CHCHD3	0.568	1.97E-08
2406420	CLSPN	0.567	2.17E-08
3474495	TRIAP1	0.567	2.13E-08
2407191	GNL2	0.566	2.37E-08
2775858	LIN54	0.566	2.43E-08
2920716	CEP57L1	0.566	2.39E-08
3888133	CSE1L	0.566	2.34E-08
2413423	TMEM48	0.565	2.55E-08
2594569	ORC2	0.565	2.52E-08
2913594	MTO1	0.565	2.56E-08
2691798	IQCB1	0.564	2.70E-08
3529156	NGDN	0.564	2.74E-08
3736290	BIRC5	0.564	2.69E-08
3766861	POLG2	0.564	2.66E-08
2955025	MRPL14	0.563	2.86E-08
2963929	RNGTT	0.563	2.89E-08
3258168	KIF11	0.563	2.84E-08
3318009	RRM1	0.562	3.23E-08
3831260	ZNF146	0.562	3.10E-08
3831514	ZNF567	0.562	3.22E-08
2499234	CCDC138	0.561	3.27E-08
2875634	ZCCHC10	0.56	3.62E-08
3151534	ATAD2	0.56	3.63E-08
2412082	FAF1	0.559	3.91E-08
2713950	ZNF141	0.559	4.04E-08
2731417	MTHFD2L	0.559	3.99E-08
2977471	ADAT2	0.559	4.09E-08
2350840	GNAI3	0.558	4.28E-08
2877314	CDC23	0.558	4.11E-08
3017123	PMPCB	0.558	4.37E-08
3448428	ASUN	0.558	4.20E-08
3629103	KIAA0101	0.558	4.37E-08
3809324	TXNL1	0.558	4.37E-08
2405364	AK2	0.557	4.59E-08
2948485	DHX16	0.557	4.47E-08
3720896	CDC6	0.557	4.67E-08
3829768	UBA2	0.557	4.69E-08
2421753	GTF2B	0.556	5.00E-08
2775965	COQ2	0.556	4.70E-08
2907596	RRP36	0.556	4.85E-08
2482211	CHAC2	0.555	5.27E-08
2580304	ORC4	0.555	5.37E-08
2633917	TRMT10C	0.555	5.20E-08
3490655	CKAP2	0.555	5.10E-08
2448971	UCHL5	0.554	5.74E-08
2807000	WDR70	0.554	5.60E-08
2979704	RMND1	0.554	5.74E-08
3817501	CHAF1A	0.554	5.67E-08
3728776	RAD51C	0.553	5.81E-08
2928392	VTA1	0.552	6.79E-08
2633460	CMSS1	0.551	7.09E-08
2836886	MRPL22	0.551	7.08E-08
2901660	PRR3	0.551	6.82E-08
3066436	PUS7	0.551	7.18E-08
2330773	CDCA8	0.55	7.67E-08
2469094	TAF1B	0.55	7.38E-08
2473571	RAB10	0.55	7.35E-08
2510485	RIF1	0.55	7.28E-08
2900372	ZNF193	0.55	7.54E-08
2695393	MRPL3	0.549	7.98E-08
2763805	DHX15	0.549	8.03E-08
2878246	PFDN1	0.549	8.02E-08
2466141	ACP1	0.548	8.33E-08

probeset	HUGO	R-value	R-pvalue
2540578	E2F6	0.548	8.72E-08
2407439	SF3A3	0.547	9.17E-08
2464499	HNRNPU	0.547	9.05E-08
2967650	RTN4IP1	0.547	9.04E-08
3011977	GTPBP10	0.547	9.58E-08
3020804	NAA38	0.547	9.41E-08
2750594	MSMO1	0.546	9.63E-08
3421177	NUP107	0.546	9.91E-08
3781429	RBBP8	0.546	9.82E-08
2434490	ENSA	0.545	1.03E-07
2512601	TANK	0.545	1.08E-07
2609560	THUMPD3	0.545	1.03E-07
2773907	SDAD1	0.545	1.05E-07
2902736	C6orf48	0.545	1.04E-07
3312490	MKI67	0.545	1.03E-07
2549455	THUMPD2	0.544	1.12E-07
2495782	LIPT1	0.543	1.21E-07
2519756	WDR75	0.543	1.26E-07
2491702	USP39	0.542	1.28E-07
2920962	FIG4	0.542	1.36E-07
3412008	PPHLN1	0.542	1.31E-07
3511189	MTRF1	0.542	1.31E-07
3840142	ZNF480	0.542	1.35E-07
2385197	GNPAT	0.541	1.44E-07
2892738	PRPF4B	0.541	1.43E-07
2984573	SFT2D1	0.541	1.38E-07
2465519	ZNF669	0.54	1.53E-07
2483016	CCDC104	0.54	1.48E-07
2485176	MDH1	0.54	1.53E-07
2869275	GIN1	0.54	1.51E-07
2967321	PREP	0.54	1.47E-07
2773655	RCHY1	0.539	1.55E-07
2972759	HDDC2	0.539	1.59E-07
2374956	TIMM17A	0.538	1.75E-07
2454532	INTS7	0.538	1.79E-07
2871717	CCDC112	0.538	1.78E-07
2913983	SENP6	0.538	1.69E-07
2766359	RFC1	0.537	1.89E-07
2962998	KIAA1009	0.537	1.86E-07
3396736	PUS3	0.537	1.92E-07
3726992	UTP18	0.537	1.80E-07
2339682	ALG6	0.536	2.02E-07
2594535	PPIL3	0.536	1.94E-07
2712906	RNF168	0.536	2.03E-07
2839671	RARS	0.536	2.00E-07
2916246	SMIM8	0.536	2.03E-07
2940551	SSR1	0.536	2.00E-07
3821908	RNASEH2A	0.536	1.96E-07
2404377	SNRNP40	0.535	2.10E-07
2406245	PSMB2	0.535	2.15E-07
2502300	DDX18	0.535	2.08E-07
2806256	DNAJC21	0.535	2.15E-07
2757319	SLBP	0.534	2.28E-07
2413180	MAGOH	0.533	2.40E-07
2651671	MYNN	0.533	2.35E-07
2675936	POC1A	0.533	2.38E-07
2961317	TMEM30A	0.533	2.36E-07
2672467	CCDC12	0.532	2.60E-07
2903343	BRD2	0.532	2.51E-07
2326410	CEP85	0.531	2.79E-07
2348854	RTCA	0.531	2.73E-07
2370991	DHX9	0.531	2.70E-07
2419235	FUBP1	0.531	2.71E-07
2936564	FGFR1OP	0.531	2.82E-07
2964771	MAP3K7	0.531	2.74E-07
2971724	FAM184A	0.531	2.81E-07
3021123	ING3	0.531	2.78E-07
3649811	NDE1	0.531	2.81E-07
2389130	EFCAB2	0.53	2.88E-07
2775562	HNRPDL	0.53	2.86E-07
2930863	PCMT1	0.53	2.97E-07
2938636	GMDS	0.53	2.97E-07
3435362	KNTC1	0.53	2.95E-07
3758317	BRCA1	0.53	2.99E-07
2695941	TOPBP1	0.529	3.15E-07
3163728	CNTLN	0.529	3.08E-07
2649609	MLF1	0.528	3.33E-07
2930592	TAB2	0.528	3.24E-07
2949431	LSM2	0.528	3.27E-07
2965674	NDUFAF4	0.528	3.33E-07
3340269	POLD3	0.528	3.27E-07
3506936	MTIF3	0.528	3.26E-07
3682135	FOPNL	0.528	3.37E-07
2608156	TRNT1	0.527	3.45E-07
3003193	CCT6A	0.527	3.59E-07
2401448	E2F2	0.526	3.85E-07
2587747	CIR1	0.526	3.77E-07
2674808	TRAIP	0.526	3.70E-07
2802739	FAM105B	0.526	3.82E-07
2855501	HMGCS1	0.526	3.87E-07
2997952	STARD3NL	0.526	3.69E-07
3050367	FIGNL1	0.526	3.74E-07
3764002	MRPS23	0.526	3.78E-07
2434319	ANP32E	0.525	3.98E-07
2908144	MAD2L1BP	0.525	4.05E-07
3560711	BAZ1A	0.525	3.99E-07
2477980	GEMIN6	0.524	4.24E-07
2519981	PMS1	0.524	4.33E-07
2580635	MMADHC	0.524	4.25E-07
3040454	TWISTNB	0.524	4.19E-07
3683806	ERI2	0.524	4.37E-07
2861952	MRPS27	0.523	4.53E-07
3374856	MRPL16	0.523	4.54E-07
2701595	DHX36	0.522	4.80E-07
3887049	UBE2C	0.522	5.06E-07
2379280	FLVCR1	0.521	5.19E-07
2420521	SSX2IP	0.521	5.36E-07
2407128	MEAF6	0.52	5.55E-07

probeset	HUGO	R-value	R-pvalue
3592366	SPATA5L1	0.52	5.64E-07
2539765	ITGB1BP1	0.519	5.87E-07
2808612	MRPS30	0.519	5.86E-07
2961816	PHIP	0.519	5.81E-07
2964200	UBE2J1	0.519	5.91E-07
3176999	RMI1	0.519	5.99E-07
3452622	RPAP3	0.519	5.85E-07
2517549	RBM45	0.518	6.24E-07
2812273	PPWD1	0.518	6.24E-07
2892979	CDYL	0.518	6.25E-07
2998404	RALA	0.518	6.36E-07
3262165	TAF5	0.518	6.33E-07
3482888	GTF3A	0.518	6.24E-07
3799542	CEP76	0.518	6.23E-07
2386397	GGPS1	0.517	6.67E-07
3388914	DCUN1D5	0.517	6.69E-07
3567469	TRMT5	0.517	6.57E-07
3804000	INO80C	0.517	6.74E-07
3884892	FAM83D	0.517	6.62E-07
3956433	CHEK2	0.517	6.66E-07
2353881	MAN1A2	0.516	7.17E-07
2422885	GLMN	0.516	6.98E-07
2478017	MORN2	0.515	7.75E-07
2517408	AGPS	0.515	7.47E-07
2845274	CCDC127	0.515	7.77E-07
2856484	MOCS2	0.515	7.47E-07
3004768	ZNF273	0.515	7.42E-07
3197528	PLGRKT	0.515	7.52E-07
3624362	LEO1	0.515	7.43E-07
4002809	APOO	0.515	7.63E-07
2452319	RBBP5	0.514	8.37E-07
2486520	ETAA1	0.514	7.86E-07
2679864	PSMD6	0.514	8.31E-07
3345593	CEP57	0.514	7.95E-07
2529782	MRPL44	0.513	8.72E-07
2834093	TCERG1	0.513	8.68E-07
2975287	HBS1L	0.513	8.42E-07
2991103	BZW2	0.513	8.89E-07
3839142	ZNF473	0.513	8.50E-07
3392840	BUD13	0.512	8.97E-07
4012949	ABCB7	0.512	9.11E-07
2406766	MRPS15	0.511	9.91E-07
2544164	C2orf44	0.511	9.77E-07
2978876	PPIL4	0.511	9.51E-07
3343008	TMEM126A	0.511	9.66E-07
3869461	ZNF616	0.511	9.86E-07
4021508	ZNF280C	0.511	9.66E-07
2412529	NRD1	0.51	1.03E-06
2476411	TTC27	0.51	1.03E-06
2828856	HSPA4	0.51	1.05E-06
2866543	CETN3	0.51	1.01E-06
3019519	IFRD1	0.51	1.03E-06
3026599	TRIM24	0.51	1.06E-06
3105467	E2F5	0.51	1.01E-06
3166718	DNAJA1	0.51	1.06E-06
3202171	PLAA	0.51	1.04E-06
3482977	POLR1D	0.51	1.03E-06
2476176	YIPF4	0.509	1.07E-06
2946232	HIST1H1C	0.509	1.12E-06
2422517	ZNF644	0.508	1.15E-06
2458017	NVL	0.508	1.19E-06
2562115	LSM3	0.508	1.13E-06
2640855	MCM2	0.508	1.14E-06
2849960	ZNF622	0.508	1.20E-06
3421630	CCT2	0.508	1.19E-06
2429277	CSDE1	0.507	1.25E-06
2521997	C2orf69	0.507	1.25E-06
3527418	PARP2	0.507	1.25E-06
3630099	TIPIN	0.507	1.24E-06
3325680	EIF3M	0.506	1.37E-06
2403470	DNAJC8	0.505	1.41E-06
2707359	DNAJC19	0.505	1.37E-06
2750227	TMA16	0.505	1.42E-06
2787005	CLGN	0.505	1.46E-06
3467315	IKBIP	0.505	1.39E-06
2586227	FASTKD1	0.504	1.49E-06
2843619	HNRNPAB	0.504	1.54E-06
2922840	KPNA5	0.504	1.47E-06
3365249	SAAL1	0.504	1.54E-06
2332144	CTPS1	0.503	1.55E-06
2498046	C2orf49	0.503	1.65E-06
2678526	C3orf67	0.503	1.64E-06
3432138	MAPKAPK5	0.503	1.60E-06
3719112	ZNHIT3	0.503	1.60E-06
2618702	ZNF620	0.502	1.66E-06
2633587	TBC1D23	0.502	1.67E-06
2784131	BBS7	0.502	1.73E-06
2868523	CHD1	0.502	1.70E-06
2894689	TMEM14C	0.502	1.72E-06
2952781	SAYSD1	0.502	1.74E-06
2962767	PGM3	0.502	1.66E-06
3638048	MRPL46	0.502	1.71E-06
2413519	HSPB11	0.501	1.81E-06
2633631	NIT2	0.501	1.76E-06
2384956	COG2	0.5	1.93E-06
2555490	XPO1	0.5	1.90E-06
2824986	COMMD10	0.5	1.95E-06
2859494	SREK1IP1	0.5	1.91E-06
3187577	CNTRL	0.5	1.93E-06
3573261	SNW1	0.5	1.86E-06
